# Culture-supported ecophysiology of the SAR116 clade demonstrates metabolic and spatial niche partitioning

**DOI:** 10.1093/ismejo/wraf124

**Published:** 2025-06-13

**Authors:** Jordan T Coelho, Lauren Teubner, Michael W Henson, V Celeste Lanclos, Conner Y Kojima, J Cameron Thrash

**Affiliations:** Department of Biological Sciences, University of Southern California, Los Angeles, CA 90089, United States; Department of Biological Sciences, University of Southern California, Los Angeles, CA 90089, United States; Department of Biological Sciences, Northern Illinois University, DeKalb, IL 60115, United States; Department of Biological Sciences, University of Southern California, Los Angeles, CA 90089, United States; Department of Biological Sciences, University of Southern California, Los Angeles, CA 90089, United States; Department of Biological Sciences, University of Southern California, Los Angeles, CA 90089, United States

**Keywords:** SAR116, heterotrophs, bacterioplankton, genomics, Alphaproteobacteria, culturing

## Abstract

Marine SAR116 bacterioplankton are ubiquitous in surface waters across global oceans and form their own order, *Puniceispirillales,* within the *Alphaproteobacteria.* To date no comparative physiology among diverse SAR116 isolates has been performed to capture the functional diversity within the clade, and further, diversity through the lens of metabolic potential and environmental preferences via clade-wide pangenomics continues to evolve with the addition of new genomes. Using high-throughput dilution-to-extinction cultivation, we isolated and genome sequenced five new and diverse SAR116 isolates from the northern Gulf of Mexico. Here we present a comparative physiological analysis of these SAR116 isolates, along with a pangenomic investigation of the SAR116 clade using a combination of metagenome-assembled genomes (MAGs, n = 258), single-amplified genomes (n = 84), previously existing (n = 2), and new isolate genomes (n = 5), totaling 349 SAR116 genomes. Phylogenomic investigation supported the division of SAR116 into three distinct subclades, each with additional structure totaling 15 monophyletic groups. Our SAR116 isolates belonged to three groups within subclade I representing distinct genera with different morphologies and varied phenotypic responses to salinity and temperature. Overall, SAR116 genomes encoded differences in vitamin and amino acid synthesis, trace metal transport, and osmolyte synthesis and transport. They also had genetic potential for diverse sulfur oxidation metabolisms, placing SAR116 at the confluence of the organic and inorganic sulfur pools. SAR116 subclades showed distinct patterns in habitat preferences across open ocean, coastal, and estuarine environments, and three of our isolates represented the most abundant coastal and estuarine subclade. This investigation provides the most comprehensive exploration of SAR116 to date anchored by new culture genomes and physiology.

## Introduction

Marine SAR116 bacterioplankton, first discovered in the Sargasso Sea [1], are ubiquitous across the epipelagic global oceans [2–4]. Although peak relative abundances may reach up to 20% of the prokaryotic community during a summer bloom [5], they are more commonly found to represent 5%–12% of the prokaryotic community [2, 5–9]. SAR116 are frequently associated with coastal phytoplankton blooms and tend to increase in abundance mid- to late-bloom [10–12]. In addition, SAR116 have been detected among marine sponge and coral samples [13–15], though the phylogenetic designations of these coral and sponge-associated representatives has yet to be resolved within the SAR116 clade. Early investigations into the evolutionary history of SAR116 delineated them as a monophyletic group within the *Alphaproteobacteria* [2, 5, 9, 13, 16], and although they were previously described as members of the *Rhodospirillaceae* [17, 18], more recently they have been classified in their own order, the *Puniceispirillales* [19, 20].

Advancements in the cultivation of marine microorganisms via high-throughput, dilution-to-extinction culturing using natural seawater media [21–23] lead to the isolation of the first three SAR116 cultured representatives [17, 18, 24], defined their aerobic chemoheterotrophic metabolism, and facilitated further genomic and physiological characterizations of the clade [17, 18]. Although extensive physiological characterizations of HTCC8037 [24] and HIMB100 [18] have yet to be published, strain IMCC1322 has received more experimental attention. Its genome encodes a green-tuned proteorhodopsin (522 nm) and light exposure enabled growth in a 20% (v/v) CO headspace compared with dark incubations where cells experienced CO toxicity [13]. More recently, IMCC1322 was shown to constitutively express proteorhodopsin regardless of the presence or absence of light [25].

Numerous investigations of *in situ* microbial assemblages have generated additional information on SAR116 physiology and spatial distributions. SAR116 have been described as active members of coastal and open-ocean systems [12] with particularly high transcriptional activity in coastal [26, 27] and estuarine waters [28]. In fact, at the Sapelo Island Microbial Observatory estuarine site, the IMCC1322 genome recruited the most transcripts of the sixteen diverse references used, implicating similar SAR116 taxa in polyphosphate and nitrate processing, and demonstrating the highest expression in genes for uptake of amino acids and five-carbon carbohydrates than other bacterioplankton [26]. In the Columbia River estuary, SAR116 were most abundant in samples with brackish salinities of 15.4 and 25.4, with high expression of metalloendopeptidases, and zinc, iron, and nickel transporters [28]. Among bacterioplankton with dimethylsulfoniopropionate (DMSP) metabolism via DMSP lyase (*dddP*) [29], two *dddP* gene operational taxonomic units (OTUs) belonging to SAR116 (OTU43 and OTU28) were the most abundant taxa, comprising 82 ± 14% of *dddP* genes in the upper euphotic zone across the NW Pacific transect [29]. This predicted physiology was experimentally validated in IMCC1322, which produced dimethyl sulfide (DMS) when supplemented with DMSP, a hallmark of DMSP lyase activity [29]. OTU43 was abundant and ubiquitous across the coastal and oligotrophic open-ocean sampling sites, whereas OTU28 was only detected in the oligotrophic open-ocean sites, suggesting biogeographic differentiation within the SAR116 clade. Overall, these *in situ* findings illuminated additional SAR116 physiologies and suggested specialized geographic distributions, indicating a much wider breadth of functional and ecological diversity than what was initially known from the first three isolates.

A previous clade-wide comparative genomic analysis classified the *Puniceispirillales* into two discrete subclades with different evolutionary histories [30]. Genomic characteristics such as GC content and intergenic spacer length suggested that one of these subclades had undergone streamlining [30] that evolutionarily selects for reductions in genome size and cell complexity [31]. This streamlined subclade was also more globally distributed across the TARA Oceans dataset [32], and its success was attributed to adaptation for a broader range of conditions relative to the non-streamlined SAR116 subclade [30]. This runs counter to canonical genome streamlining theory that predicts evolution towards a specialist rather than a generalist phenotype [31, 33, 34]. Nevertheless, these data demonstrated large-scale evolutionary and habitat heterogeneity within the SAR116 clade and generated many new questions about the life history and microdiversity within this group.

Our recent isolation of five new SAR116 representatives [7, 35], coupled to the reconstruction of hundreds of new SAR116 metagenome-assembled genomes (MAGs) [36], offered an opportunity to explore physiological diversity and update previous observations of the *Puniceispirillales*, especially related to the known boundaries of SAR116 functional diversity. To contextualize our isolates within the *Puniceispirillales*, and to more comprehensively investigate the functional diversity and niche partitioning across the clade, we employed comparative physiology, microscopy, and genomics with new (n = 5) and previously sequenced (n = 2) SAR116 isolate genomes, MAGs (n = 258), and single-amplified genomes (SAGs, n = 84) for a total of 349 *Puniceispirillales* genomes. Here, we have found the SAR116 clade to be composed of three distinct subclades with further subclade structure leading to 15 subclades in total. We predict that differentiation among subclades was driven by habitat preferences and metabolic niche partitioning, including osmolyte synthesis and transport, vitamin and amino acid synthesis, and sulfur oxidation metabolisms. Physiological characterization of our SAR116 isolates demonstrated their salinity preferences, along with phenotypic variation in temperature ranges and cell morphologies. These results expand our understanding of *Puniceispirillales* functional diversity and enable us to better constrain the ecological niches of subgroups within the SAR116 clade.

## Materials and methods

### Isolation, genome sequencing, and assembly

We isolated new SAR116 strains using high-throughput dilution-to-extinction cultivation and identified them via 16S rRNA gene comparisons as described (Table S7) [7, 35]. Genomic DNA sequencing and library preparation were completed at the University of Southern California (USC) Molecular Genomics Core using the NextSeq 550 system (Illumina). Additionally, we performed Oxford Nanopore long-read sequencing in house on four of the five genomes. We generated hybrid assemblies with Unicycler v0.4.8 [37], and used SPAdes v3.13.1 [38] for our Illumina-only assembly. Assembled genomes were assessed for quality and completeness using CheckM [39]. Details of isolation, sequencing, quality checking, and assembly, are in Supplemental Text [38, 40, 41].

### Additional taxon selection

We combined our five SAR116 isolate genomes with all publicly available SAR116 genomes from the Genome Taxonomy Database (release89) [19, 20], Global Ocean Reference Genomes Tropics [42], and the OceanDNA MAG catalog [36]. Quality assessment, completion, and genome dereplication were done as previously described [43, 44]. We required all genomes to have <5% contamination, MAGs to have ≥70% completion, and SAGs to have ≥50% completion to remain in the analysis, resulting in a total of 349 SAR116 genomes (Table S1). Outgroup taxa (n = 31) for phylogenomic analysis were selected from cultured representatives across various orders of *Alphaproteobacteria* as described in Muñoz-Gómez et al. 2019 [45] and assessed for quality, completeness, and dereplicated as described above.

### Phylogenomics

We completed phylogenomic analysis in Anvi’o [46] v7.1 as previously described [43, 44]. Amino acid sequences were retrieved using “anvi-get-sequences-for-hmm-hits” using the “—Rinke_et_al” HMM profile [47]. Genes were trimmed with TrimAl [48] and aligned with MUSCLE [49], both with default settings, then concatenated using geneStitcher [50]. We used IQ-TREE v2.1.2 [51] for maximum-likelihood inference using traditional bootstrapping with 1000 replicates, and the automated amino acid substitution best-fit model estimator “-m MFP” which selected LG + R10 as the best model. We visualized the resulting tree using *Ggtree* v3.2.1 and *Treeio* v1.18.1 R packages [52–54], rooted at the midpoint, and nodes were ordered in increasing order.

### Pangenomics and metagenomic recruitment

We conducted pangenomic analysis in Anvi’o [46] v7.1 similarly as reported [44], generating the pangenome annotation summary using *anvi-summarize,* and annotation lists for sulfur oxidation analyses can be found in Table S2. Additionally, we used KEGG Decoder and Expander [55] to identify diversity in physiological and metabolic gene pathways, modified to include the osmolyte table in Henson et al. [56], along with additional genes associated with vitamin, organic carbon, amino acid, and metal transport. Specific genes accession numbers can be found in Table S3. With the output data we predicted subclade physiologies by calculating the median pathway completion values for each subclade. We visualized the output data using custom R scripts (on FigShare—https://doi.org/10.6084/m9.figshare.29002031). Metagenomic recruitment was done as previously described [43, 44]. Reads-Per-Kilobase-Mapped (RPKM) was used as a proxy for subclade abundance and calculated using RRAP [57]. Additional details on these analyses, including statistical evaluations of habitat distribution, are in Supplemental Text [58–64].

### Single-gene phylogenetics

We generated a 16S rRNA gene phylogeny comparison with previous 16S rRNA subclade designations [13], and to investigate the coral and sponge association among SAR116 members [65]. Sulfur oxidation gene phylogenies were also generated to evaluate the evolutionary histories of these proteins within SAR116. Additional details in Supplemental Text [66–74].

### Temperature and salinity tolerances.

To test the temperature and salinity tolerances of cultured representatives, three LSUCC SAR116 isolates—LSUCC0719, LSUCC0744, and LSUCC0684—were inoculated from 1 ml cryostocks into 5 ml of our complex and defined artificial seawater medium, MWH2 [35] (Table S4) using 10 ml sterile borosilicate glass test-tubes (#1512, Globe Scientific, New Jersey, USA). A range of seven temperatures and five salinities were tested. Growth for all experiments was measured with a BD Accuri C6 Plus flow cytometer (BD, New Jersey, USA) using 1x SYBR Green as described [7, 43] and plotted using sparse-growth-curve [75]. Additional details on experimental design in Supplemental Text.

### Scanning electron microscopy

We inoculated three LSUCC SAR116 isolates, LSUCC0719, LSUCC0744, and LSUCC0684, from cryostocks (1 ml) into 100 ml of MWH2 medium [35] (Table S4) in sterile 125 ml polycarbonate flasks (FPC0125S, TriForest, Irvine, CA), and fixed them at a final concentration of 2.5% (v/v) glutaraldehyde (G5882, Sigma-Aldrich) after growth to mid-exponential phase. Scanning electron microscopy (SEM) was performed at the USC NanoImaging Center, and SEM images were analyzed in ImageJ [76] to quantify cell size measurements. Additional details on SEM image prep and SEM image analysis in Supplemental Text.

## Results

### New genome characteristics

**Figure 1:**
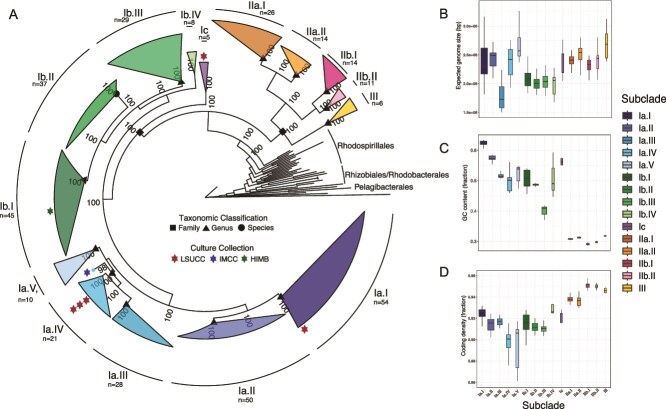
Subclade structure and genome characteristics across the SAR116 clade. (A) Phylogenomic tree of the SAR116 clade with other members of the *Alphaproteobacteria* as an outgroup. The scale bar represents 0.1 changes per position. Bootstrap support values (n = 1000) are indicated at nodes. Red stars next to subclades indicate isolate representation and the color designates the culture collection the isolate is housed in according to the key inside the tree. Shapes overlaying internal nodes of SAR116 subclades represent the taxonomic classification of the descendants in the subclade according to the key inside the tree. Panels B-D indicate the estimated genome size (B), GC content (C), and coding density (D) (estimated via CheckM) of SAR116 genomes by subclade.

We sequenced and assembled five new SAR116 genomes spanning a wide diversity within subclade I (Fig. 1A). Four out of five isolate assemblies produced closed, circular genomes, and all had 0% estimated contamination (Table 1). CheckM estimated the genome of LSUCC0684 to be 97.98% complete, however the genome assembled into a single circular contig, and thus the LSUCC0684 genome invites re-evaluation of the appropriate single-copy marker genes designated for SAR116 in CheckM. The LSUCC isolate genomes ranged in size from 2.59–2.80 Mbp with a G + C content range of 51%–58%, and a predicted protein coding gene range of 2448–2658. The full set of SAR116 genomes all encoded a single rRNA operon and had estimated genome sizes ranging from 1.76–3.95 Mbp (mean 2.25 Mbp) (Fig. 1B), G + C contents spanning 29–63% (Fig. 1C), and coding densities from 81% to 95.7% (Fig. 1D), all of which represented extremely large intraclade ranges.

**Table 1:** Assembly statistics for new LSUCC SAR116 isolate representative genomes generated in this study. Genome size estimates were calculated by dividing the assembly size by the estimated percent completion (fraction) output from CheckM.

	Completion (%)^*^	Contamination (%)	Actual/Estimated size	Contigs assembled	GC content (fraction)	Predicted genes	rRNA operon number
LSUCC0719	99.13	0.00	2,793,311 bp	43 N50: 535962	0.58	2658	1
LSUCC0226	100	0.00	2,678,285 bp	1 N50: 2678285	0.51	2536	1
LSUCC0396	100	0.00	2,592,803 bp	1 N50: 2592803	0.51	2428	1
LSUCC0744	100	0.00	2,653,841 bp	1 N50: 2 635 841	0.51	2485	1
LSUCC0684	100	0.00	2,704,644 bp	1 N50: 2 704 644	0.55	2551	1
Other SAR116	50.74 – 99.94 Avg: 79.98	0.00 – 4.99 Avg: 1.92	1.51–3.95 Mbp Avg: 2.25 Mbp	1–1048 Avg # contigs: 363	0.29–0.63 Avg: 0.49	1055–2875 Avg: 1999	1

### Phylogenetic diversity

**Figure 2:**
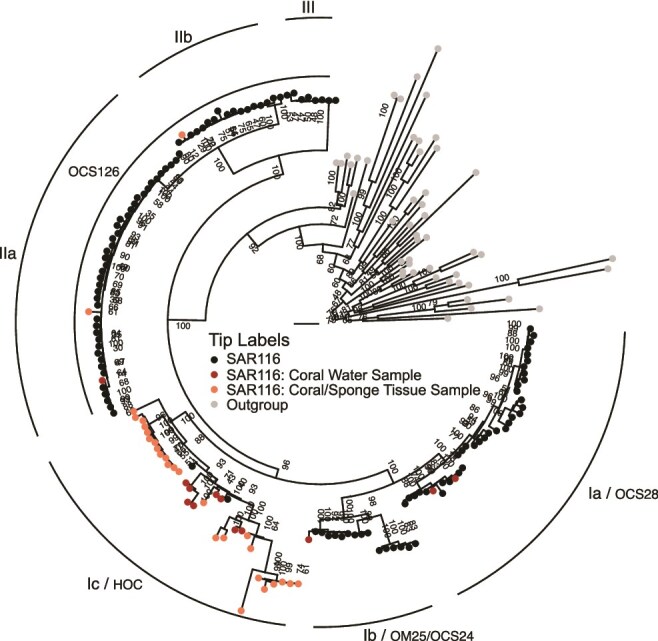
16S rRNA gene phylogenetic tree of SAR116 members and outgroup members of the *Alphaproteobacteria*. The scale bar represents 0.3 changes per position. Bootstrap values (n = 1000) are represented at nodes. Black tip labels indicate SAR116 members, red tip labels indicate SAR116 members from water sampled at the coral: water interface, orange tip labels indicate SAR116 members from ground coral tissue samples, and gray tip labels indicate outgroup members. Subclades are labeled with the updated subclade designations from this investigation (underlined) and the historical 16S rRNA gene subclade designations.

Our updated phylogeny, using 380 total genomes (SAR116 n = 349, Outgroup n = 31) and average amino acid identity (AAI), supported dividing the SAR116 clade into three major subclades (I, II, and III) with additional structure defining 15 subclades in total (Fig. 1A, Fig. S1, Table S5). This updated phylogeny recovered the same major subclade structure established via 16S rRNA genes [13] (Fig. 2, S2), and a similar structure to a previous phylogenomic inference [30], however we have defined a third major subclade and have also expanded on the known diversity of subclade I, previously denoted as the high GC group (HGC) [30]. Here, our updated subclades II and III comprise the previously defined low GC (LGC) subclade [30]. 16S rRNA gene identity between the two most divergent genomes in the SAR116 clade (Fig. S1) was 86.98% via BLAST [60], and pairwise AAI was 52.47%, both supported an Order level divergence [61]*,* matching the GTBD classification. The *Puniceispirillales* have been previously proposed to comprise four families [30], however we conservatively confirmed the presence of two families using AAI taxonomic classification [62]. One family corresponds to subclade I and the other to subclades II and III (Fig. 1A). Furthermore, we assigned putative genus demarcations to 12 of the 15 subclades (Fig. 1A). The newly assembled genomes LSUCC0226, LSUCC0396, and LSUCC0744 branched within subclade Ia.IV, LSUCC0719 in subclade Ia.I, and LSUCC0684 in subclade Ic (Fig. 1A). Thus, our five new isolates belonged to three separate genera that spanned the diversity of subclade I.

### Host-association

Although SAR116 is a cosmopolitan marine surface group [77–79], it has also been previously detected in marine sponge and coral tissues [14, 15, 65, 80], with relative abundances up to 10% of the prokaryotic endosymbiont community [65]. We investigated subclade affiliations of the putative endosymbiont SAR116 members in the context of our revised taxonomy to understand evolutionary origins of these taxa. The overwhelming majority of coral/sponge-associated SAR116 16S rRNA gene sequences grouped with subclade Ic, with only two host-associated sequences branching in subclade II (Fig. 2). This suggests a specific endosymbiotic host-associated divergence within the *Puniceispirillales*. However, the presence of non-host associated sequences in the Ic subclade, one from the LSUCC0684 isolate genome and the other from the AG-917-F20 SAG collected at the Bermuda Atlantic Time Series [42], supports the assertion that host-associated *Puniceispirillales* have a planktonic life stage [80]. Regardless, the current evidence suggests that host-association does not occur commonly within the SAR116 clade and is primarily restricted to subclade Ic.

### New metabolic observations

**Figure 3:**
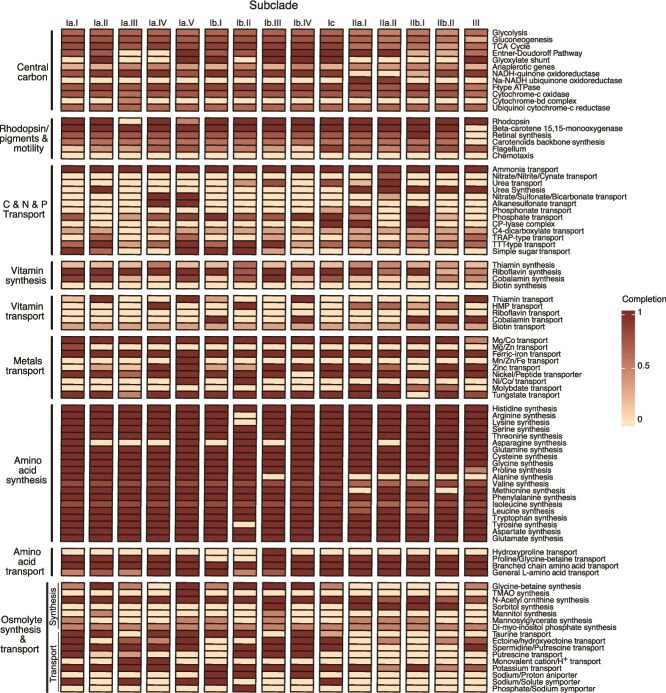
Output from KEGG-decoder modified to include osmolyte synthesis and transport genes along with additional metals and vitamin transporters. Each row is the median pathway representation by subclade, and every column is a metabolic pathway. The darker the color the more complete the pathway, with the pale peach color indicating no detection and dark red indicating a complete pathway.

We compared gene content in SAR116 to delineate differences in metabolic potential across subclades. Broadly, we recapitulated previous observations that *Puniceispirillales* genomes have predicted capacity for gluconeogenesis, the TCA cycle, but incomplete glycolysis (via Embden-Meyerhoff-Parnas) pathways [17, 18, 30] (Fig. 3). And, similar to previous results [13, 17, 18] carbon monoxide oxidation genes were present throughout the SAR116 clade (Table S6). Proterhodopsin, the light-mediated proton-pump, has been previously described in SAR116 [13]. Here we found it to be prolific across the clade, however in our updated phylogeny, subclade Ia.III does not encode for the protein (Fig. 3). There was no genetic evidence of carbon fixation or carbon dioxide assimilation, nor was there evidence of anaerobic respiration, thus supporting previous findings of an obligate aerobic and heterotrophic physiology [13, 18, 30]. Below are our predictions of SAR116 metabolic potential that have changed based on our updated analysis with new closed isolate genomes, and/or have not been previously discussed in the most recent SAR116 pangenomics analysis [30].

Trace metal transport potential, important for metalloenzyme function and activity, was diverse across the SAR116 clade, with few systems conserved in all subclades (Fig. 3). We predicted that SAR116 cells predominantly rely on ferric iron transport through the *afuABC* complex to meet their iron needs. One subclade, Ia.V, carries a manganese/zinc/iron transporter (*sitABCD)*, that may also transport ferrous iron. Other metals that appeared important based on widely conserved transporters were magnesium and/or cobalt (all but subclade III encoded for a magnesium/cobalt transporter, *corC*), and tungsten (via *tupABC* is present in all subclades except IIb.I). Nickel may also be important for SAR116. The nickel/cobalt transporter, *rcnA*, was not widely distributed (only in subclades Ia.V and Ib.II). However, all but subclade Ib.IV had the nickel/peptide transporter, *ABC.PE.SPP1* (Fig. 3), so subclades may obtain nickel via different systems. More sparsely distributed were transporters for zinc and manganese (subclades Ia.IV*–*V, Ib.III–IV, Ic, Ia.I, Ib.II, and III had *znuABC*; only subclade Ia.I had the manganese/zinc transporter *ABC.ZM.SAP*), as well as molybdate (*modABC* was only in subclade II) (Fig. 3). Although it remains possible that the absence of some of these transporters may result from the lack of complete genomes, the cases where a transporter was missing in all genomes from a subclade likely indicates a true gene absence. The differential distributions of these metal transporters across SAR116 offer potential insight into how the subclades are interacting with metals, and how trace metal scarcity can limit metalloenzyme activity and overall physiology. Namely, subclade Ia.V enocodes the most metal transporters of all SAR116 subclades, possibly indicating a diverse trace metals requirement among their proteome relative to the other SAR116 subclades. Additionally, even though TupABC has a higher affinity for tungstate, it also transports molybdate [81]; thus the supplementary molybdate transport from *modABC*, unique to subclade II, suggests an elevated requirement for molybdate in that group.

Vitamin synthesis and transport was also differentiated across the SAR116 subclades (Fig. 3). Most subclades contained genomes with evidence of riboflavin synthesis, and several subclades had genomes with genes for cobalamin synthesis. Some subclades that lacked complete cobalamin synthesis appeared to compensate with cobalamin transport (Ib.I, Ib.IV, II.bI, II.bII, III). Many subclades had transport genes either for thiamin or the thiamin precursor, 4-amino-5-hydroxymethyl-2-methylpyrimidine (HMP), but not both. Some had neither (Fig. 3). We did not find evidence of biotin synthesis genes in most genomes, but there were potential biotin transporters in most subclades. Consistent with this hypothesis, our culture medium supplied all of the above vitamins, aside from HMP (Table S4). We predict that all of our LSUCC SAR116 isolates require the exogenous biotin supplied through the culture media as there is no evidence for biotin synthesis genes. LSUCC0719 (Ia.I) encoded genes to synthesize riboflavin and cobalamin, but did not encode a full pathway for thiamin synthesis (Fig. S3). However, LSUCC0719 encoded a full transport protein complex for thiamin, therefore we expect that it was transporting exogenous thiamin supplied in the culture medium. LSUCC0744 (Ia.IV) carried a complete biosynthesis pathway for riboflavin, and a partial pathway for thiamin (Fig. S3). It did not encode a transport complex for thiamin, but did encode a full protein complex for HMP transport, which could be present because HMP is a common contaminant of commercially produced thiamin [82]. LSUCC0684 carries genes to synthesize riboflavin and cobalamin (Fig. S3), and it encodes a full protein complex for thiamin transport; thus, we expect that LSUC0684 was transporting exogenous thiamin supplied in the culture medium.

Using marker genes for amino acid biosynthesis, we predicted that SAR116 subclades could make most amino acids, with some notable exceptions (Fig. 3). We did not find evidence for alanine synthesis in subclade Ib.III genomes, nor in any genomes of subclades II and III, all of whom were missing alanine dehydrogenase, involved in the reversible conversion of pyruvate to alanine [83]. Alanine synthesis in SAR116 was predicted to be exclusively carried out via alanine dehydrogenase as no SAR116 genome encoded alanine transaminases [84] (Table S6). Similarly, we did not find evidence for methionine synthesis via homocysteine methyltransferase in subclade IIb.II and in a little more than half of the genomes in subclade IIa.I (Fig. S3). The average genome completion of 70% for subclade IIa.I (Fig. S4) leaves open the possibility that the inconsistent presence/absence of homocysteine methyltransferase may result from incomplete genomes. We also predicted asparagine auxotrophy in Ia.II and Ia.III genomes as asparagine synthase was undetected (Fig. S3). However, in subclades Ia.IV, Ib.I, Ib.III, and IIa.II, we found a putative asparagine synthase across a subset of genomes (Fig. S3). Although this pattern could also result from incomplete genomes, the closed isolate genomes in subclade Ia.IV reflected the same inconsistency, with no predicted asparagine synthases in LSUCC0396 or LSUCC0226, but one in LSUCC0744 (Fig. S3). However, because we did not provide asparagine or alanine in our culture medium (Table S4), either these organisms do not require these amino acids in all cases, or asparagine and alanine synthesis are completed by other proteins. We did supply methionine (Table S4), therefore we predict that our isolates used the exogenous methionine from the culture medium.

**Figure 4:**
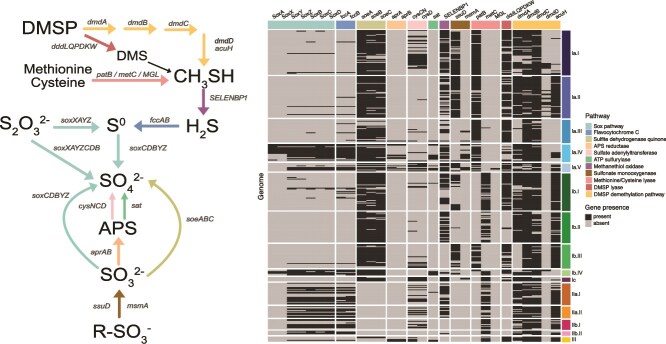
Sulfur oxidation gene heatmap and metabolic pathway map. To the left, the metabolic pathway map, reaction arrow colors match metabolic pathway color represented in the gene presence and absence heatmap to the right. Every row represents a genome, grouped by subclade, and every column represents a sulfur oxidation gene, grouped by pathway. Gray boxes indicate the gene to be absent and black boxes indicate the gene to be present.

DMSP is an important metabolite in SAR116 sulfur metabolism that can support heterotrophic growth, with genes for both cleavage and demethylation pathways [13, 29, 30]. Our results fill in a previously missing step in the demethylation pathway and links DMSP demethylation to potential for chemolithotrophic sulfur oxidation (Fig. 4). Similarly to these prior studies, we found DMSP demethylation via *dmdABC* encoded across the clade, except for subclade Ib.III, in which *dmdA* was missing from all genomes (Fig. 4). The *dmdD* gene*,* encoding an Enoyl-CoA hydratase (ECH) that synthesizes methanethiol and completes the DMSP demethylation pathway, was sparsely distributed across the SAR116 clade (only in subclades Ia.I, Ia.II, and Ib.IV). However, other ECHs such as acryloyl-CoA hydratase (*acuH)* have a broad substrate specificity and can act on methylthioacryloyl-CoA, the substrate for *dmdD,* and thus *dmdD* and *acuH* are functionally redundant in DMSP catabolism [85–87]. We found that *acuH* genes were more widely dispersed throughout SAR116 than *dmdD*, occurring in subclades Ia.II, Ia.IV, IbI-III, II and III. Therefore, our research predicts that all subclades except Ia.III and Ib.III can carry out complete DMSP demethylation either via *dmdABCD* or *dmdABC*/*acuH* (Fig. 4) [88, 89]. Furthermore, we predict that production of methanethiol via DMSP demethylation could also ultimately serve to generate hydrogen sulfide in some subclades of SAR116 via methanethiol oxidase (SELENBP1/*mtoX*) and thereby supply an electron donor for chemolithotrophic sulfur metabolism (Fig. 4). We found predicted methanethiol oxidases in all but subclades Ib.III, IIb.I, and III. This matches recent evidence that this protein is widespread in marine bacteria, including Roseobacter [90], and methanotrophs [91]. Thus, most SAR116 may be able to salvage energy from methanethiol by converting it to hydrogen sulfide [90], rather than shunt it into amino acid metabolism, when needed. In contrast, DMSP lyase (encoded by *dddL, Q, P, D, K,* and/or *W),* a carbon acquisition strategy that releases dimethyl sulfide (DMS) [92], was only detected in subclades I and III and not at all in subclade II. We also found no evidence that SAR116 carry *dmoAB* to convert DMS to methanethiol (Fig. 4). Therefore, it is probable that DMSP cleavage to DMS through DMSP lyase is a dead-end for this pathway in SAR116 [93].

SAR116 harbor a variety of genes involved in sulfur oxidation [30, 94]. The potential for thiosulfate oxidation to sulfate via the SOX pathway has been previously highlighted [30], however, in we found that the majority of *SOX*-encoding SAR116 genomes did not encode a complete pathway, specifically, we found no evidence of *soxAX* in any subclade II or III genomes (Fig. 4). Only members from subclades Ia.IV and Ib.IV had the full pathway (*soxXAYZCDB*), including the *soxAX* genes whose protein complex binds thiosulfate [95]. In subclades II and III, and a few genomes across subclade I, the gene suite comprised *soxCDBYZ*, that likely oxidizes elemental sulfur or other sulfane sulfur species to sulfate, and may also oxidize sulfite to sulfate [95]. Thus, not all *SOX*-containing SAR116 genomes had the potential to oxidize thiosulfate. In addition, the SAR116 members that carried the predicted *soxCDBYZ* gene suite, along with other members of the SAR116 clade, encoded for flavocytochrome C (*fccAB*) that oxidizes hydrogen sulfide to elemental sulfur (Fig. 4). Therefore, in subclades II and III, hydrogen sulfide may provide the sulfur source that ultimately gets oxidized to sulfate via *soxCDBYZ*, which is significant for predicting interactions with sulfur compounds and intracellular sulfur cycling.

We also predicted that some SAR116 can oxidize sulfite to sulfate using the sulfite dehydrogenase quinone, *soeABC* (Fig. 4). Aside from two genomes in subclade III, we found this gene suite exclusively in subclade I. Sulfonates that undergo conversion to sulfite via a sulfonate monooxygenase (*ssuD, msmA*) [96, 97] likely supply sulfite to the *soeABC* complex. The *msmA* gene has been previously highlighted in SAR116 [30], however without including *ssuD*, an accurate distribution of sulfonate metabolism is lost because subclade I exclusively encodes either SsuD or MsmA (Fig. 4). Both proteins act on methanesulfonate, however, SsuD can also act on alkanesulfonates [96]. Together, these predicted metabolisms point towards sulfonates as important organosulfur compounds that SAR116 subclade I may be metabolizing to glean additional energy through lithotrophic sulfite oxidation.

The extensive presence of sulfur oxidation genes in SAR116 prompted us to examine their evolutionary history. The key sulfur oxidation genes *soxB* and *fccB* show evidence of vertical transmission throughout the SAR116 clade, whereas *soeA* may have been horizontally transferred extensively throughout subclade diversification (Supplemental Text, Figs. S19–S21).

### Habitats

**Figure 5:**
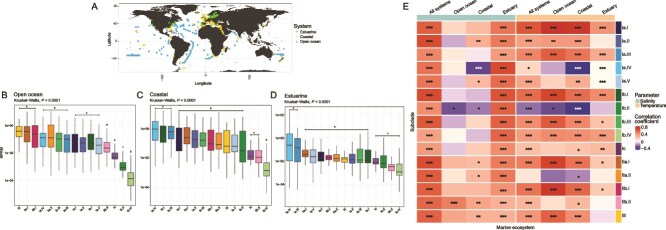
Relative abundance of subclades by ecosystem from metagenomic recruitment of 1059 metagenomes to all 349 SAR116 genomes. A) World map showing the spatial distributions of the metagenomic samples used for recruitment to the SAR116 genomes. Every data point is a metagenomic sampling site, and marine environments are represented by color. Metagenomes were classified as Open Ocean (B), coastal (C), or estuarine (D). Subclades are ordered from the most abundant to least abundant subclade based on median log10-transformed RPKM values. RPKM = reads per kilobase of genome per million mapped reads. Statistical groupings for subclades that share similar mean values are reported above boxplots in panels B-D. (E) Pearson correlation heatmap of subclade RPKM values against both salinity and temperature across metagenomes. Columns are separated into either salinity or temperature, and each column represents a different marine ecosystem (all systems, open ocean, coastal, or estuarine). Subclades are represented in rows. Blue boxes indicate a negative Pearson correlation, and red boxes indicate a positive Pearson correlation. “^*^” = *P* value <0.05, “^**^” = *P* value <0.01, “^***^” = *P* value <0.001.

Recruitment of reads from 1049 marine metagenomes to the 349 SAR116 genomes showed that SAR116 subclades were euphotic-zone organisms with different habitat preferences according to the three marine environments: estuarine, coastal, and open ocean (Fig. 5A). We rarely detected SAR116 below the euphotic zone (Table S9), similar to previous findings [5, 30, 98], therefore we decided to focus on surface ocean biogeography across major marine ecosystems. Subclade III was the dominant group in open ocean systems, followed by subclade II and then subclade I (Fig. S5). Spatial distributions at the major subclade level only showed differences in open ocean systems (Fig. S5), however when we looked closer at the distributions of groups within subclades I and II, there were clear habitat preferences (Fig. 5, Table S8). Subclades III, IIa.I, and IIb.I dominated open ocean systems, and the remaining subclades broke into multiple groups with decreasing relative abundance (Fig. 5B). In contrast, subclades Ia.IV, Ib.I, and Ia.III were the most abundant groups in coastal systems (Fig. 5C). Finally, in estuarine systems, subclades Ia.III and Ia.IV (for which we have three isolates) were the dominant SAR116 subclades (Fig. 5D). Thus, habitat diversity in SAR116 mainly occurs at or around the genus level.

Because we observed specific subclades that were enriched in coastal and estuarine systems where salinity can be a primary differentiator of microbial communities, we compared osmolyte synthesis and transport genes within SAR116 (Fig. 3), as the distribution of these genes can provide hypotheses about how prokaryotes mitigate osmotic stress [56, 99, 100]. SAR116 genomes carried genes to synthesize the non-protein amino acid N-∂-acetyl-ornithine, and sugar derivative di-*myo*-inositol phosphate, both demonstrated to serve as osmolytes under increasing extracellular salt conditions [101, 102]. These organisms could also likely uptake potassium via the *trk* potassium transport system, another common strategy for osmotic stress among prokaryotes [102]. The above pathways were conserved across the SAR116 clade (Fig. 3) and likely represent core osmotic stress strategies for SAR116.

Some osmolyte genes appeared related to habitat differentiation among the subclades. Subclade I, which comprised groups that were more abundant in coastal and estuarine areas, encoded nearly twice as many pathways related to osmotic stress response compared to subclades II and III (Fig. 3). Such a genetic expansion could indicate that these microorganisms are better adapted to salinity fluctuations, and therefore explain their greater abundances in coastal/estuarine habitats, a pattern we have observed previously related to brackish water-adapted SAR11 strains [44]. Furthermore, subclade I had a unique enrichment of ionic symporters/antiporters (Fig. 3). Members of subclade Ib carried a sodium/proton antiporter and interspersed across subclade I was a sodium/solute symporter. We found a monovalent cation/H^+^ antiporter exclusively in the genomes of the most abundant subclades in estuarine environments, subclades Ia.III and Ia.IV. Each of these ionic symporters/antiporters have roles in managing osmotic stress [103–105], and the monovalent cation/H^+^ antiporter has been proposed as conferring an adaptive response to salinity fluctuations in estuaries [28].

**Figure 6:**
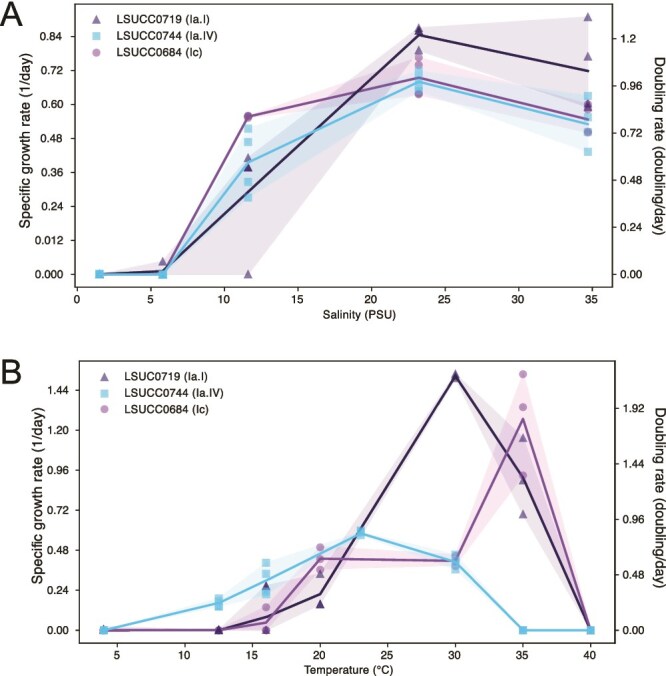
Phenotypic variation of SAR116 LSUCC isolate representatives across temperature and salinity ranges. LSUCC0719, representing subclade Ia.I in dark blue. LSUCC0744, representing subclade Ia.IV in light blue. LSUCC0684, representing subclade Ic in purple. A) Salinity vs. growth rate. B) Temperature vs. growth rate.

The few genes encoded by subclades II and III to mitigate osmotic stress were primarily involved in de novo synthesis of osmolytes rather than rapid uptake from the environment (Fig. 3). This strategy occurs in *Vibrionaceae* in high salinity when extracellular osmolytes are limited [44, 56, 100], and is a hallmark of freshwater SAR11 with restricted salinity distributions [56], and thus represents a potential strategy in the oligotrophic open ocean for subclade II. Specifically, subclade II harbored genes for synthesizing an additional sugar derivative, sorbitol. Subclade III, however, had a balanced representation of synthesis and transport of osmolytes, a strategy seen among other halotolerant microbes [56, 106]. In addition to the aforementioned clade-wide strategies, subclade III are also predicted to synthesize glycine-betaine, along with the transport of ectoine and spermidine/putrescine (Fig. 3), potentially serving as additional osmoprotectants or as carbon and nitrogen sources [99, 107]. Overall, the varied strategies to mitigate osmotic stress among SAR116 subclades highlight potential physiological responses and adaptations to environmental stressors throughout SAR116 evolution.

In addition to location, we evaluated the relationships between salinity, temperature, and subclade abundance. Broadly, SAR116 subclade abundances were positively correlated with salinity (Fig. 5E, S6), especially in estuarine systems (Fig. 5E, S7), however salinity did not correlate well with subclade abundances in coastal (Fig. S8) or open ocean (Fig. S9) environments, potentially due to the small ranges in salinity among these sites. In contrast, SAR116 subclades appeared more differentially adapted to temperature (Fig. 5E, S10). Subclades Ia.I, IIa.I, and IIb.I abundances were most positively correlated with temperature, with the strongest correlations in coastal (Fig. S11) and open ocean (Fig. S12) environments. Subclades Ia.IV, Ib.II, and IIa.II were negatively correlated with temperature (Fig. 5E, S10–S13), suggesting potentially cold-adapted ecotypes. In estuarine systems, there were no strong correlations between subclade abundances and temperature (Fig. S13). Subclades Ia.III, Ia.IV, and Ib.I, the most abundant subclades in coastal systems (Fig. 5C), had different relationships to temperature in coastal systems; where subclades Ia.III and Ib.I were positively correlated with increasing temperature, subclade Ia.IV was negatively correlated with increasing temperature (Fig. S11), suggesting that subclades Ia.III and Ib.I are the dominant SAR116 coastal subclades in warmer regions, and subclade Ia.IV in cooler regions. Collectively, this indicated that temperature may drive SAR116 spatial distributions and habitat preferences.

### Isolate physiology and morphology

We characterized key growth parameters for three isolates representing the breadth of cultured SAR116 diversity: LSUCC0719 (Ia.I), LSUCC0744 (Ia.IV), and LSUCC0684 (Ic). All could grow in salinities ranging from 11.6–34.7 ppt and had no discernable differences in growth rate across this range, with optimum growth occurring at 23.2 ppt (Figs. 6A, S14). However, each isolate had a specific temperature tolerance range and optimum temperature for growth (Figs. 6B; Table 2; Fig. S15). These isolates all had maximum growth rates under the conditions tested between roughly one and two doublings per day (Table 2).

**Table 2:** Salinity and temperature ranges, optima, and growth rates across LSUCC SAR116 isolate representatives.

	Salinity range (PSU)	Optimal salinity (PSU)	Temperature range (°C)	Optimal temperature (°C)	Maximum growth rate (doublings • day^−1^)
LSUCC0719	11.6–34.7	23.2	16.0–35.0	30.0	2.2 $\pm$ 0.04
LSUCC0744	11.6–34.7	23.2	12.5–30.0	23.0	0.98 $\pm$ 0.09
LSUCC0684	11.6–34.7	23.2	22.0–35.0	35.0	1.83 $\pm$ 0.89

**Figure 7:**
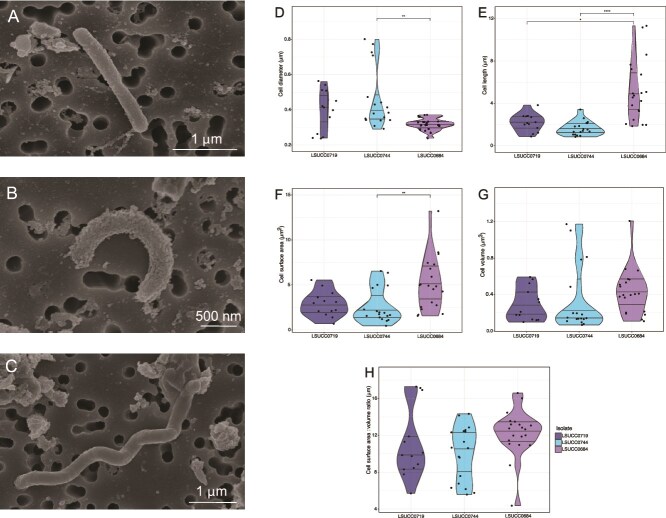
Cell sizes and morphologies for SAR116 isolates, LSUCC0719 (n = 13 cells), LSUCC0744 (n = 18 cells), LSUCC0684 (n = 20). Measured cell diameters (A), cell lengths (B), cell surface areas (C), cell volumes (D), and cell surface area:volume ratios. Representative SEM images for LSUCC0719 (F, scale bar = 1$\mu$m), LSUCC0744 (G, scale bar = 500 nm), LSUCC0684 (H, scale bar = 1$\mu$m). “^*^” = *P* value <0.05, “^**^” = *P* value <0.01, “^****^” = *P* value <0.0001.

Our SAR116 isolates each displayed different morphologies, covering bacilli, (LSUCC0719, Fig. S16), vibrio (LSUCC0744, Fig. S17), and spirilla (LSUCC0684, Fig. S18) (Fig. 7F-H, Table S10). Although their morphologies were different, LSUCC0719 and LSUCC0744 showed no significant differences in their cell dimensions (Fig. 7A-E), whereas LSUCC0744 and LSUCC0684 had differences in cell diameter (Fig. 7A) and surface area (Fig. 7C). The greatest variation in cell dimensions occurred across cell lengths (Fig. 7B), where LSUCC0684 had a considerable range between 1.85–11.32 $\mu$m (Fig. S2). Nevertheless, despite the variation in morphologies and cell sizes, there were no significant differences in cell volume (Fig. 7D), or cell surface area to volume ratios (Fig. 7E) between these cells, suggesting a conservation of these characteristics.

We also cataloged extensive pleomorphy that may be related to a complex life cycle in the host-associated (Ic) representative, LSUCC0684 (supplemental text). These cells appeared both as long spirilla, but also as flattened large cocci (Fig. S22B-D). We also captured an image of LSUCC0684 undergoing a considerable morphological change from the spirillum to the cocci form (Fig. S22A). Thus, we believe these organisms are pleomorphic and the image of the cell spanning both morphologies may be a visualization of the conversion to the endosymbiotic calcifying SAR116 cells found in marine sponges [80], termed “calcibacteria”. These endosymbiotic “calcibacteria” SAR116 cells were more spherical in shape, ~1$\mu$m in diameter, in sponge tissues [80]. This matches our measured diameters of the spherical LSUCC0684 morphology ranging from 0.72–1.25 $\mu$m in diameter (Fig. S22B-D). Some appeared to be hollowed or collapsed (Fig. S22B). We hypothesize that this morphological transformation occurs as LSUCC0684 cells enter stationary phase, which may correspond to a life cycle change where they are taken up by sponge hosts [80].

## Discussion

Our isolation of new, diverse SAR116 strains motivated us to investigate the *Puniceispirillales* in the context of new genomes and the first comparative phenotypic data for the group. In addition to five new strains in three putative genera, we contributed the first four closed SAR116 genomes and used them to anchor the largest SAR116 pangenomic study to date. These closed genomes allowed us to make more confident predictions of auxotrophy, and our study updated SAR116 phylogeny, provided an updated description of metabolic heterogeneity within the clade, and the first evidence for subclade-specific environmental diversification.

We found that a notable difference in SAR116 subclades was temperature differentiation. We observed this both within environmental abundances, via metagenomic recruitment (Fig. 5E), and corroborated some of these patterns through experimentation with our isolates (Fig. 6B). Subclade Ia.I positively correlated with temperature across all marine systems, particularly in open ocean and coastal systems, and the representative isolate strain LSUCC0719 also had a temperature growth optimum of 30°C. Similarly, our warmest-adapted isolate, LSUCC0684 (temperature growth optimum of 35°C), belonged to subclade Ic, which were also positively correlated with temperature in coastal and estuarine systems. This group is also the subclade predicted to have a host-associated life cycle, and thus the 35°C temperature optimum aligns with endosymbiotic relationships with corals and sponges [65, 80] where host temperature tolerances can range 23-32°C [108]. Conversely, our more cold-adapted strain, LSUCC0744, belonged to subclade Ia.IV, which was negatively correlated with temperature. The temperature range for LSUCC0744 also matched that of the previously isolated Ia.IV strain IMCC1322 (12.8–34.2^ο^C) [13]. Maximum growth rates of the LSUCC SAR116 cultured representatives indicate they fall in the spectrum between the canonical slow growth of SAR11 [56], and faster growers such as members of the Oligotrophic Marine Gammaproteobacteria (OMG) [109]. Without cultured representatives from subclades II and III, we do not definitively know their growth temperature ranges, however our data suggested that they are positively correlated with increasing temperature, particularly the members of subclades II and III. Although these open-ocean groups may be resilient to increasing sea-surface temperatures, the dominant coastal and estuarine subclade Ia.IV that prefers cooler temperatures, may face displacement. Thus, SAR116 contributions to coastal and estuarine biogeochemistry may be altered in a changing climate. For example, subclade Ia.IV was one of the only groups predicted to carry out complete thiosulfate oxidation. With thiosulfate representing a key intermediate of sulfur cycling within marine microbial communities [110, 111], displacement of this abundant subclade may disrupt sulfur cycling in coastal and estuarine systems.

Isolate physiology also supported the assertion that the dominant coastal and estuarine subclade Ia.IV was adapted to lower salinities. Strain LSUCC0744 had an optimum growth salinity of 23.2 ppt, which corroborates previous reports from the other Ia.IV isolate strain IMCC1322, that had an optimum of 28 ppt [13] Our other isolates, all belonging to the larger subclade I, also had the same optimum salinity (Fig. 6B). Subclade I comprised groups that dominated in coastal and estuarine systems, so these salinity optima corroborate those patterns. We now know from our distribution data (Fig. 5) that obtaining subclade I isolates was more likely than those from subclades II or III in the coastal sampling locations yielding the LSUCC, HIMB, and IMCC cultures [7, 13, 18, 35]. This predicted (Fig. 5E) and observed (Fig. 6B) preference for brackish waters in subclade I may result from the wide array of osmotic stress response systems encoded by subclade I genomes (Fig. 3), that likely improve tolerance to salinity fluctuations in coastal and estuarine systems [112], particularly through rapid uptake of positive and negative ions [100].

We can use our new characterizations of habitats and metabolic preferences to contextualize previous ecological observations of SAR116. These organisms were most abundant in the Columbia River estuary system among brackish salinities between 15.4 and 25.4, and showed high expression of metallopeptidases, along with zinc, iron, and nickel transporters [28]. Phenotypic responses (Fig. 6B), salinity correlations (Fig. 5E), and habitat preferences (Fig. 5C&D) suggest that these metabolically active SAR116 members were from subclade I. Our pangenomics results even helped us define these estuarine representatives further as members of subclade Ia.IV or Ia.V because these Ia groups carried zinc, nickel, and iron transporters (Fig. 3). Separately, SAR116 were the most abundant DMSP lyase-carrying (*dddP*) taxa in a northwest Pacific transect, separated into two *dddP* OTUs with differing biogeographical patterns: OTU43 was abundant in both coastal and open-ocean sites and OTU28 was only detected in the open-ocean [29]. We found no evidence for *dddP* in subclade II (Fig. 3) and given the preference of subclade III for open-ocean systems (Fig. 5B) compared to the coastal/open-ocean subclade I habitats (Fig. 5B&C), we predicted OTU43 to be a member of subclade I and OTU28 to be a member of subclade III. Indeed, two blastn alignments of the *dddP* sequences (n = 168 771 sequences) from that study against the LSUCC0719 and AG_899_J15 genomes (representing subclades I and III, respectively) showed that our subclade I representative had 545 *dddP* sequence hits, with 19 sequences greater than 90% identical, whereas the subclade III representative had 44 *dddP* sequences hits with only one sequence greater than 90% identical. Thus, subclade I likely dominated the *dddP*-containing SAR1116 population in this region.

DMSP cleavage is just one of several types of sulfur metabolism that were differentially distributed across the SAR116 clade (Fig. 4). Different groups can access a variety of sulfur compounds which likely links them to phytoplankton in distinct ways. Sulfonates are produced by numerous phytoplankton groups in coastal and open ocean systems [113]. Sulfonate monooxygenase (*ssuD, msmA*) genes that convert sulfonates to sulfite were encoded exclusively by subclade I (Fig. 4), suggesting that sulfonates are a phytoplankton-supplied source of sulfite for these organisms. Additionally, even though most SAR116 have the genetic capability to demethylate DMSP and generate sulfide from methanethiol, only subclades I and IIa had the genetic capability to oxidize hydrogen sulfide derived from DMSP via methanethiol oxidase (Fig. 4). Thus, we hypothesize subclades I and IIa interact with both the organic and inorganic sulfur pools via multiple organic sulfur species likely originating from phytoplankton. Hydrogen sulfide in marine surface waters is usually quite low (< 1 nM) [114] but has roughly a 24 hour half-life for abiotic oxidation [115]. In anoxic systems where redox chemistry supports higher production of hydrogen sulfide, the half-life of sulfide can be ~15 minutes [116, 117], and thus biotic oxidation of sulfide can outcompete abiotic oxidation. Therefore, SAR116 may oxidize extracellular hydrogen sulfide via flavocytochrome C (*fccAB*), and we also hypothesize that hydrogen sulfide can be produced intracellularly through L-cysteine metabolism [118, 119] or through DMSP metabolism via oxidation of methanethiol [90]. For subclades that encode *fccAB* (that can produce elemental sulfur from hydrogen sulfide) (Fig. 4), if that elemental sulfur is not immediately oxidized to sulfate (via *soxBCDYZ)*, then it may accumulate intracellularly as sulfur inclusions for later oxidation [120, 121]. Investigation of potential sulfur storage in subpopulations of SAR116 warrants further investigation, as this may confer an advantage to these organisms, particularly in the oligotrophic open oceans and act as a sulfur sink in the surface ocean, analogously to Candidatus *Thioglobus* (a.k.a. SUP05) Gammaproteobacteria [122].

Cell size is a critical component in determining the relative contributions of microbial cells to biogeochemical cycles, and this work provides a new, more comprehensive view of morphological diversity and cell dimensions for SAR116. Previous data showed considerable variation in cell morphology across two different isolates, IMCC1322 (Ia.IV) [13] and HIMB100 (Ib.I), suggesting that SAR116 may display a wide range of cell shapes and sizes. We corroborate the vibrioid shape of subclade Ia.IV with LSUCC0744 (Fig. 7B), and both isolates have similar cellular dimensions. Strain HIMB100 (Ib.I) had an extended spirillum morphology with cells ranging 1–5$\mu$m in length [18], more similar to that of LSUCC0684 (Ic; Fig. 7C). Conversely, strain LSUCC0719 had a simpler bacillus shape compared to the other cell types. Thus, within just subclade I, there were at least three distinct morphologies across a range of sizes. LSUCC0719 and LSUCC0744 cells were large relative to other cultivated marine microbes like members of SAR11 [44] or OM252 [43], but were more similar in size to members of the *Roseobacter* lineage [123]. The conserved surface area to volume ratio, despite varied sizes (particularly cell length, Fig. 7E) and morphologies, suggests that SAR116 might regulate cell size relative to this ratio, and/or this characteristic was inherited from their shared common ancestor because of its importance for nutrient acquisition [92].

The range in cell length for LSUCC0684 was large (Fig. 7E, Fig. S2), and may therefore represent a strain capable of filamentous growth, similarly to sulfide-oxidizing *Beggiatoa spp.* [124]. LSUCC0684 displayed significant pleomorphy, so we hypothesize that filamentous growth may occur in preparation for the next stage in the LSUCC0684 life cycle where cells increase in size prior to undergoing a morphological transformation (Fig. S18), potentially in anticipation for uptake by corals and sponges [65, 80]. Endosymbiotic *Proteobacteria* in marine sponges aid in larval settlement, a process regulated by nitric oxide signaling [125] via arginine biosynthesis that stimulates the sponge host to produce nitric oxide and thus initial larval settlement [126]. Subclade Ic were prototrophic for arginine (along with all other amino acids- Fig. 3), and therefore amino acid production may be a role for endosymbiotic SAR116 Ic cells in marine sponges. Further work should explore the involvement and activities of SAR116 Ic in sponge and coral holobionts. Other endosymbiotic lineages, such as squid endosymbiont *Vibrio fischeri* and jellyfish endosymbiont *Symbiodinium microadriaticum* [127], each have planktonic phase of their life-cycle [128, 129]. Therefore, at least transient endosymbiotic relationships of what we consider “free-living” organisms may be occurring more frequently than previously thought.

Altogether, this investigation demonstrated SAR116 diversification through metabolic and spatial niche partitioning, anchored by information from new and diverse SAR116 isolates. Our experimental results expanded the known salinity and temperature tolerances across subclade I and corroborate culture-independent assessments of their habitat preferences and spatial distributions. Our cultures also revealed large differences in cell dimensions and shapes throughout subclade I and identified a consistency of surface area:volume ratios despite large differences in cell sizes. We were also able to identify a peculiar morphological change in the subclade Ic representative, LSUCC0684, that may link these organisms to a host-associated part of their life cycle. This isolate provides new opportunities to investigate symbioses of SAR116 with animals, a very understudied aspect of SAR116 biology. The diversity of SAR116 leaves open many other avenues for investigation: future research to validate the hypothesized sulfur oxidation metabolisms, osmotic stress regulation, and putative vitamin and amino acid auxotrophies will be important to deepen our understanding of how SAR116 physiology influences sulfur cycling, interacts with other members of the microbial community, and differentiates subclades in relationship to salinity variation.

## Supplementary Material

supplementary-material_wraf124

## Data Availability

Assembled isolate genomes for LSUCC0226, LSUCC0396, LSUCC0684, LSUCC0719, and LSUCC0744 are available on NCBI under CP166132, CP166131, CP166130, JBFPJN000000000, and CP166129, respectively. Raw reads are available under BioProject PRJNA1133775. Supplemental files, including phylogenetic files, R scripts, and the pangenome summary (Table S6), are available on FigShare (https://doi.org/10.6084/m9.figshare.29002031). Cultures of the LSUCC isolates used in this analysis are available upon request.
