# Supplementary material for: Culture-supported ecophysiology of the SAR116 clade demonstrates metabolic and spatial niche partitioning
Source: ISME J. 2025 Jun 13;19(1):wraf124. doi: 10.1093/ismejo/wraf124 (PMC12262181; doi:10.1093/ismejo/wraf124)
Supplement: supplementary-material_wraf124 [file supplementary-material_wraf124.zip › Coelho_Supplemental_text_v6_wraf124.docx]

Supplemental Information for ***Culture-supported ecophysiology of the SAR116 clade demonstrates metabolic and spatial niche partitioning***

Jordan T. Coelho^1^, Lauren Teubner^1^, Michael W. Henson^2^, V. Celeste Lanclos^1^, Conner Y. Kojima^1^, J. Cameron Thrash^1,*^

^1^Department of Biological Sciences, University of Southern California, Los Angeles, CA, 90089, USA

^2^Department of Geophysical Sciences, University of Chicago, Chicago, IL, 60637

^*^Correspondence: thrash@usc.edu

**Methods**

*Isolation, genome sequencing, and assembly*

We isolated five SAR116 strains - LSUCC0226, LSUCC0396, LSUCC0719, LSUCC0744, and LSUCC0684 - using high-throughput dilution-to-extinction cultivation and identified them via 16S rRNA gene comparisons, as previously described [[1]](https://paperpile.com/c/H8ueyx/soFa4). LSUCC0226 was isolated on October 1, 2015 from the Calcasieu Jetties in Cameron, LA (29.760164 -93.340159), and LSUCC0396 on April 6, 2016 off of Freshwater City Road in Vermilion Parish, LA (29.53084, -92.326147), from the both using JW2 media [[1]](https://paperpile.com/c/H8ueyx/soFa4). LSUCC0684 and LSUCC0719 were isolated on August 22, 2016 from the Calcasieu Jetties in Cameron, LA (29.760164 -93.340159), and LSUCC0744 was isolated on November 11, 2016 off of Freshwater City Road in Vermilion Parish, LA (29.53084, -92.326147) each using MWH2 media [[2]](https://paperpile.com/c/H8ueyx/DpQhS) (Table S4, Table S7). For genome sequencing, we grew these isolates axenically in a range of 500 mL – 1.5 liters of MWH2 medium from cryostocks [[2]](https://paperpile.com/c/H8ueyx/DpQhS). Cells were filtered onto 0.2 µm polyethersulfone (PES) filters (Pall Corporation, New York, USA) and stored at -20˚C for genomic DNA extraction. We extracted genomic DNA using the GenElute Bacterial Genomic DNA Kit (Sigma-Aldrich, Missouri, USA), then cleaned and concentrated the DNA with the Zymo Genomic DNA clean and concentrator kit. Library preparation for NextSeq 550 (Illumina) sequncing was done via a KAPA HyperPrep kit, and sequencing was performed at the University of Southern California (USC) Molecular Genomics Core. To obtain additional DNA for Oxford Nanopore sequencing, we grew separate cultures of LSUCC0226, LSUCC0396, LSUCC744, and LSUCC0684 from cryostocks in 1 L MWH2. DNA extraction, cleaning, concentrating, and quantification was done as described above. DNA was sheared to a 20 kbp fragment size using Covaris g-TUBE (Covaris, Inc. Massachusetts, USA). Quality assessment of the sheared DNA was done at the USC Molecular Genomics core using the Agilent 4200 TapeStation. Library preparation began with 700 ng of DNA per isolate strain and was performed using a modified 1D Native barcoding genomic DNA (with EXP-NBD103 and SQK-LSK108) protocol. Prior to sequencing, we checked the pore activity of the R9.4.1 FLOMIN106 flow cell and configured it using the MinKNOW 3.1.19 software. We loaded 300 ng of pooled DNA onto the flow cell, and sequenced with a MinION for 20 hours generating 174.26 k reads and 811.18 Mbp. Prior to assembly, Nanopore reads were base-called using Guppy v330 (Oxford-Nanopore Technologies) with GPU configuration to increase base-calling speed, and a minimum quality score of 7.0. We demultiplexed Nanopore reads using Porechop [[3]](https://paperpile.com/c/H8ueyx/frCGj) under the stringent binning settings, with the additional option to remove barcode adapters in the middle of the read. Illumina reads were first trimmed using Trimmomatic v0.38 [[4]](https://paperpile.com/c/H8ueyx/8cad2) to remove bases less than 20 on a Phred-33 scale. Leading and trailing bases of a read were removed if below the threshold of 20, and bases were cut if the average quality score fell below 20 on a sliding window of 13 bases. Additionally, a read was removed if it was below 40 bp in length. We hybrid-assembled genomes that received both Illumina and Nanopore sequencing using Unicycler v0.4.8 [[5]](https://paperpile.com/c/H8ueyx/Qjuu0) on default settings. The genome of LSUCC0719 only received Illumina sequencing and we assembled the trimmed reads using SPAdes v3.13.1 [[6]](https://paperpile.com/c/H8ueyx/gY3Mf) with the automatic coverage cutoff specified. We assessed genome assembly quality and completeness using CheckM v.1.1.3 [[7]](https://paperpile.com/c/H8ueyx/m883j) using lineage_wf settings for both hybrid and Illumina-only assemblies.

*Genomic Relatedness*

We used average nucleotide identity (ANI) and average amino acid identity (AAI) to delineate SAR116 subclade structure and to assess the relatedness of subclade members. We computed ANI across SAR116 members using FastANI [[8]](https://paperpile.com/c/H8ueyx/oNxzy), and computed pairwise AAI across SAR116 members using CompareM [[9]](https://paperpile.com/c/H8ueyx/85yUB), both with default settings. To determine the divergence of the overall clade, the 16S rRNA gene sequences from the two most divergent genomes were compared via BLAST [[10]](https://paperpile.com/c/H8ueyx/SGRGW). We evaluated the relationship between 16S rRNA gene identity, phylogenomic tree topology, and AAI to confirm clade structure and ultimately determined subclade delineations and taxonomic classifications with AAI [[11](https://paperpile.com/c/H8ueyx/iGfLv), [12]](https://paperpile.com/c/H8ueyx/MPnvM). Taxonomic classification AAI threshold values followed the standards as previously proposed [[12],](https://paperpile.com/c/H8ueyx/MPnvM) 45-65% AAI represent the same family, 65-95% AAI represent the same genus, and 95-100% represent the same species.

*Environmental preferences*

To understand the relationship between SAR116 subclades and major marine environments we performed metagenomic read recruitment to 1,059 metagenomes spanning a range of marine, coastal, and estuarine environments as previously described [[13, 14]](https://paperpile.com/c/H8ueyx/CCRUg+jgYID), using Reads-Per-Kilobase-Mapped (RPKM) as a proxy for subclade abundance. RPKM values were calculated for each individual genome using RRAP [[15]](https://paperpile.com/c/H8ueyx/WzgsE), and then RPKMs for all the genomes in a subclade were summed to obtain subclade RPKM. We classified the sample sites across the 1,059 metagenomes [[14]](https://paperpile.com/c/H8ueyx/jgYID) as Open Ocean, Coastal, or Estuarine. A metagenome was considered “Coastal” if it was collected from within the distance of the local coastal shelf and considered “Estuarine” if it was collected within an estuary. All other metagenomes were considered to be “Open Ocean”. Salinity from sites associated with metagenomic data was reported as unitless, as these measurements arise from a conductivity ratio. We used a Kruskal-Wallis test [[16]](https://paperpile.com/c/H8ueyx/Ijz2C) to determine if there were statistically significant differences between SAR116 subclade abundances and salinity of the metagenomic samples. To quantify pairwise subclade abundance differed based on salinity values, a Dunn’s test [[17]](https://paperpile.com/c/H8ueyx/mjyJ5) was employed. The statistical results of the described tests can be found in Table S8. Subclade abundance data based on marine environment was visualized using custom R-scripts made in R-Studio [[18, 19]](https://paperpile.com/c/H8ueyx/sqlIe+6mkYU). The metagenomic recruitment data included in Figure 3a-e can be found in Table S9, which also includes sampling depth in meters. For metagenomes where sampling depth was not included in the metadata, we confirmed the metagenome was sampled from surface water (0.5-1.5m)[[20–23]](https://paperpile.com/c/H8ueyx/qCSH+G6LZ+8ACl+kuYK).

*Single Gene Phylogenetics*

We compared our updated SAR116 taxon selection to previously described SAR116 16S rRNA gene subclade structure using 16S rRNA genes extracted from the Anvi’o database via ‘anvi-get-sequences-for-hmm-hits’ and sequences from the 16S rRNA gene phylogenetic tree in Lee et al. 2019 [[24]](https://paperpile.com/c/H8ueyx/BnaQx). To investigate SAR116 association with corals and sponges, we additionally obtained 16S rRNA genes in Bonthond et al. 2018 [[25]](https://paperpile.com/c/H8ueyx/2TNbC) from the SILVA database [[26–28]](https://paperpile.com/c/H8ueyx/UzEeN+HBNst+baJqm). Sequences were trimmed with TrimAl [[29]](https://paperpile.com/c/H8ueyx/ZlubR) and aligned with MUSCLE [[30]](https://paperpile.com/c/H8ueyx/k0dbM), both with default settings. Maximum-likelihood inference was done using IQ-TREE v2.1.2 [[31]](https://paperpile.com/c/H8ueyx/UoHjI) using the automated nucleic acid substitution best-fit model estimator ‘-m MFP’ and traditional bootstrapping with 1000 replicates. The selected nucleic acid substitution model was TVMe+R5, and the resulting tree was visualized using *Ggtree* v3.2.1 and *Treeio* v1.18.1 R packages [[32–34]](https://paperpile.com/c/H8ueyx/siy73+YFi6C+ouaZS), rooted at the midpoint, and nodes were ordered in increasing order.

We used SoxB within the thiosulfate oxidizing SOX pathway for phylogenetic inference as it is the gene responsible for releasing sulfate at the end of the pathway, and is the marker gene for full thiosulfate oxidation [[35]](https://paperpile.com/c/H8ueyx/uVizX). The active site encoding subunit, f*ccB* in the flavocytochrome c (*fccAB)* complex [[35]](https://paperpile.com/c/H8ueyx/uVizX), and the active site encoding subunit, *soeA*, within the sulfite dehydrogenase quinone (*soeABC)* complex [[36]](https://paperpile.com/c/H8ueyx/0H3De) were also chosen for the phylogenetic inference of sulfide oxidation and sulfite oxidation, respectively. Amino acid sequences for all genomes were generated in Anvi’o v7.1 using ‘anvi-get-sequences-for-gene-calls’. For each sulfur oxidizing gene, the corresponding amino acid sequences were searched against the NCBI RefSeq database [[37]](https://paperpile.com/c/H8ueyx/mu45D) via BLAST [[10]](https://paperpile.com/c/H8ueyx/SGRGW). The top 25 best-hit were retained for each query, and then further filtered to remove any duplicates. This resulted in 166 SoxB, 313 FccB, and 672 SoeA amino acid sequences for phylogenetic inference of sulfur oxidizing genes. Sequences were aligned, trimmed, and phylogenetic inference and visualization was completed as above. The amino acid substitution models were LG+I+G4, LG+G+R6, LG+R9 for SoxB, FccB, and SoeA, respectively.

*Temperature, and Salinity Tolerances*

We tested the growth ranges and optima for temperature and salinity using one isolate from each of the three phylogenetic groups for which we had cultures. Although we have five SAR116 isolates, LSUCC0744 was chosen as the sole representative of IIa.I because it shares > 98% 16S rRNA gene identity with LSUCC0226 and LSUCC0396. To assess the temperature growth ranges across these isolates, each isolate representative was inoculated from late-exponential phase in triplicate into 5 mL of MWH2 in 10mL sterile borosilicate glass test-tubes (#1512, Globe Scientific, New Jersey, USA) and incubated in the dark at 4, 12, 16, 22, 30, 35, and 40^ο^C. Salinity growth ranges were also tested across these isolates, and similarly, each isolate representative was inoculated from late-exponential phase in triplicate into 5 mL of modified MWH artificial seawater medium with salinities ranging from 1.5 - 34.7 ppt (MWH1 - MWH5) into 10mL sterile borosilicate glass test-tubes, and incubated in the dark at 23^ο^C. Salinity for the growth experiments was calculated based on the chlorinity (salinity (ppt) = 1.80655 x Cl (ppt) [[38]](https://paperpile.com/c/H8ueyx/aIMXw) of the “base salts”. Growth for all experiments was measured with the BD Accuri C6 Plus flow cytometer (Becton, Dickinson and Company, New Jersey, USA) using 1x SYBR Green as described [[1, 13]](https://paperpile.com/c/H8ueyx/soFa4+CCRUg) and growth rates were calculated using sparse-growth-curve [[39]](https://paperpile.com/c/H8ueyx/7t6Xg). To contextualize these phenotypic responses to salinity and temperature, we compared the metagenomic recruitment to the summed RPKMs for each isolate clade based on salinity and temperature across all metagenomic datasets. We fit linear regressions based on salinity or temperature vs. subclade log-transformed RPKM and visualized the results using “ggplot2” [[40]](https://paperpile.com/c/H8ueyx/u9lRW). The MWH2 culture medium recipe can be found in Table S4.

*Scanning electron microscopy*

Three LSUCC SAR116 isolates – LSUCC0719, LSUCC0744, and LSUCC0684 -were inoculated from cryostocks (1 mL) in 100 mL of MWH2 media [[2]](https://paperpile.com/c/H8ueyx/DpQhS) in sterile 125 mL flasks (FPC0125S, TriForest, Irvine, CA), and fixed at a final concentration of 2.5% (v/v) glutaraldehyde (G5882, Sigma-Aldrich) after reaching exponential growth. Scanning electron microscopy (SEM) was performed at the USC NanoImaging Center. SEM preparation was performed as described in [[14]](https://paperpile.com/c/H8ueyx/jgYID). Briefly, we filtered cells through a 0.2 µm PES filter (Pall Corporation, New York, USA) and adhered cells to the filter membrane via a poly-L-lysine coating. Cell membranes were stained with 0.1 M HEPES buffered 0.05% Ruthenium Red (RuRed) and 10% sucrose and incubated with cells for 10 minutes. The stain was fixed into cells by a 0.1 M HEPES buffered 0.05% RuRed, 0.8% Osmium tetroxide, and 10% sucrose solution and incubated for 25 minutes. After, we washed the filter membrane with 0.1 M HEPES and 10% sucrose, followed by 10 mL of deionized water. We then dehydrated the filters sequentially at 50%, 70%, 95% and 100% ethanol, then stored in 100% ethanol until imaging.The resulting SEM images were analyzed in ImageJ [[41]](https://paperpile.com/c/H8ueyx/YSVKf) to quantify cell size measurements.

Cell volume and cell surface area measurements were modeled based on the specific morphology of the cell. Bacillus morphologies, due to their resemblance to a capsule, were modeled using Equations 1 & 2, which are equations for modeling volume and surface area of a capsule (Figure S1). Vibrio morphologies were modeled using Equations 3 & 4 for curved rod-shaped cells, or 5 & 6 for horseshoe shaped cells. Curved rod-shaped cells were modeled to emulate two hemispheres (Figure S16), and horseshoe shaped cells were modeled to emulate one cylinder with two hemispheres at either end (Figure S17). Spirillum morphologies were modeled using Equations (7 & 8), where every helix was modeled as a cylinder and each end of the cell was modeled as a hemisphere, and overall surface area and volume were a sum of the number of helices plus the cell ends (Figure S18). In Equations 1-8, the variable *h* represents the length of the capsule minus the radius to to capture the dome volume and surface area at the end of a capsule. In Equations 7 & 8 the variable *n* represents the variable total number of helices per cell.

Equation (1): Volume, Bacillus

$$\pi r^{2}(\frac{4}{3}r) + h$$

Equation (2): Surface area, Bacillus

$$2\pi r(2r+h)$$

Equation (3): Volume, Vibrio (curved rod)

$$(\frac{2}{3}\pi r^{3})x2 + (\pi r^{2}h)x2$$

Equation (4): Surface area, Vibrio (curved rod)

$$(2\pi r^{2})x2 + (2\pi rh)x2$$

Equation (5): Volume, Vibrio (horseshoe)

$$(\frac{2}{3}\pi r^{3})x2 +\pi r^{2}h_{1} + \pi r^{2}h_{2} + \pi r^{2}h_{3}$$

Equation (6): Surface area, Vibrio (horseshoe)

$$(2\pi r^{2})x2 + 2\pi rh_{1} +2\pi rh_{2} + 2\pi rh_{3}$$

Equation (7): Volume, Spirillum

$$(\frac{2}{3}\pi r^{3})x2 +\pi r^{2}h_{1} + \pi r^{2}h_{2} + \pi r^{2}h_{3} + ... \pi r^{2}h_{n}$$

Equation (8): Surface area, Spirillum

$$(2\pi r^{2})x2 + 2\pi rh_{1} +2\pi rh_{2} + 2\pi rh_{3} + ... 2\pi rh_{n}$$

We tested statistical differences in cell measurements by first employing a Bartlett test [[42]](https://paperpile.com/c/H8ueyx/zMaNa) to check for equal variance in the cell measurements. If variance was unequal between cell measurements, then a Welch’s ANOVA [[43]](https://paperpile.com/c/H8ueyx/mqZfi) was performed to determine if the difference between the mean cell measurements was statistically significant. If variance was equal, then a standard ANOVA was performed [[44]](https://paperpile.com/c/H8ueyx/b1Exz). Finally, we used a Dunn’s test [[17]](https://paperpile.com/c/H8ueyx/mjyJ5) to determine pairwise differences between cell measurement means. Cell measurements (Table S10) and statistics were visualized using “ggplot2” [[40]](https://paperpile.com/c/H8ueyx/u9lRW) and “rstatix” [[45]](https://paperpile.com/c/H8ueyx/SdGh9).

**Results**

*New metabolic predictions*

In addition to an aa3-type cytochrome-c oxidoreductase, two isolates, LSUCC0744 (Ia.IV) and LSUCC0684 (Ic), also had predicted high-oxygen-affinity oxidoreductases (cytochrome c oxidase: cbb3-type and cytochrome bd complex, Table S6), though these genes were not found among other subclade Ia.IV or Ic representatives, which either indicates that our isolates are unique compared to their close relatives, or that we were able to detect these genes only because we had complete genomes. Members of subclade Ia.III only encoded subunit II of the cytochrome bd complex, and although subunit I can function without subunit II [[46]](https://paperpile.com/c/H8ueyx/ANHu7), it is unclear if the inverse is true. Alternatively, a novel protein may serve as an analogous substitution for subunit I. Although the overall SAR116 clade abundance typically declines with decreasing oxygen [[47]](https://paperpile.com/c/H8ueyx/fjOyR), the relationship between subclade Ia.III and low oxygen has yet to be established. Regardless, the presence of these high-affinity oxidoreductases indicates the potential for SAR116 aerobic metabolism under low oxygen conditions.

Methanethiol oxidase, which transforms methanethiol to hydrogen sulfide, is another gene predicted to supply organic sulfur substrates for downstream inorganic sulfur oxidation (Figure 4). Aside from DMSP (discussed above), methanethiol may also come from methionine or cysteine via the *patB/metC* cysteine ligase, or *MGL* methionine ligase, both of which have shown to form thiols [[48]](https://paperpile.com/c/H8ueyx/ahudf), and are involved in methionine salvage pathways [[49]](https://paperpile.com/c/H8ueyx/FZQfR). SAR116 genomes almost exclusively carry either *patB, metC,* or *MGL*, suggesting an analogous metabolic function among SAR116 subclades. Aside from subclades Ib.III, IIb.II, and III, methanethiol oxidases are present throughout the rest of the SAR116 subclades (Figure 4). Subclades that do not harbor *fccAB* to oxidize hydrogen sulfide to elemental sulfur likely utilize hydrogen sulfide for cysteine and methionine biosynthesis [[50]](https://paperpile.com/c/H8ueyx/sgtKP).

*Evolutionary history of sulfur oxidizing genes in SAR116*

SAR116 formed a monophyletic group within the SoxB phylogenetic tree (Figure S19) that was sister to the *Rhizobiales* and *Rhodospirillales*, similarly to the 16S rRNA gene and phylogenomic trees (Figure 1a), suggesting a vertical inheritance of these genes. An unclassified *Rhodobacteraceae* sequence branched within the SAR116 clade and was most closely related to SAR116 IIb.I, indicating a potential misclassification, or horizontal gene transfer. Subclades I and II formed distinct clades, and their branching structures did not exactly match that of the updated phylogenomic tree (Figure S1), implying horizontal transfer from the *Rhizobiales* to SAR116 subclade Ia.IV, that then diversified with SAR116 evolution. Many of the outgroup SoxB sequences belonged to known sulfur oxidizers [[51–53]](https://paperpile.com/c/H8ueyx/Df7Ie+BHBjc+Q118E), providing stronger evidence for the metabolic potential of thiosulfate oxidation within SAR116.

SAR116 members formed a monophyletic group within the phylogenetic tree of FccB (which oxidizes hydrogen sulfide to elemental sulfur) (Figure S20). SAR116 are sister taxa to *Rhizobiales,* many of which are known sulfur oxidizers [[54]](https://paperpile.com/c/H8ueyx/adR2A), similar to many other prokaryotic taxa in the *fccB* phylogenetic tree [[35, 55]](https://paperpile.com/c/H8ueyx/uVizX+yhTsR), which validates the metabolic potential of hydrogen sulfide oxidation with SAR116. Like the SoxB phylogeny, the monophyletic structure and branching patterns suggest that *fccB* was horizontally transferred from the *Rhizobiales* to members of subclade I, then further diversified throughout SAR116 evolution

The SAR116 SoeA (for oxidation of sulfite to sulfate) phylogeny had a polyphyletic structure (Figure S21), unlike the SoxB and FccB phylogenies. Group 1 in the tree, comprised of members from subclades Ia, Ib, and III, were sister taxa to the *Rhodospirillales*; and Group 2, comprised of subclades Ib.IV, Ic, and a member from subclade Ia.IV, were sister to members of the *Rhodobacterales* and *Rhizobiales*. The observed paraphyletic structure indicates that this metabolism has had a different evolutionary path for subclades Ib.IV and Ic than subclades I and III. Additionally, the branching patterns within Groups 1 and 2 are different from the phylogenomic tree (Figure S1), indicating a horizontal gene transfer from the *Rhodospirillales* in Group 1 and the *Rhizobiales* in Group 2. Both members of the SAR116 sister taxa are known sulfur oxidizers which validates this metabolic potential in SAR116.

**Supplemental Figures**

**Figure S1: Phylogenomic tree of the SAR116 clade with other members of the *Alphaproteobacteria* as an outgroup (left), and pairwise Average Nucleotide Identity (ANI) vs. Average Amino acid Identity (AAI) of the SAR116 clade (right).** The scale bar on the phylogenomic tree represents 0.1 changes per position, and bootstrap support values (n=1000) are indicated at nodes. Shapes overlaying internal nodes indicate taxonomic classification where squares represent Family, triangles represent Genus, and circles represent Species. Stars next to tip labels represent isolates based and the color of the star indicates the culture collection they are housed in - red (LSUCC), blue (IMCC), and green (HIMB). Dashed lines delineate subclades within the SAR116 clade and their corresponding ANI v. AAI values. Red circles represent genomes that received pairwise 16S rRNA gene alignment, and pairwise AAI comparison to validate the Order level divergence of the SAR116 clade. Accession numbers and/or identifiers for all genomes are provided in Table S1.

**Figure S2: 16S rRNA gene phylogenetic tree of SAR116 members and outgroup members of the *Alphaproteobacteria*.** The scale bar represents 0.3 changes per position. Bootstrap values (n=1000) are represented at nodes. Black tip labels indicate SAR116 members, red tip labels indicate SAR116 members from water sampled at the coral:water interface, orange tip labels indicate SAR116 members from ground coral tissue samples, and gray tip labels indicate outgroup members. Subclades are labeled with the updated subclade designations from this investigation (underline) and the historical 16S rRNA gene subclade designations.

**Figure S3: KEGG-Decoder output heatmap for metabolic pathway completion.** Every row genome that corresponds to phylogenetic ordering based upon the phylogenomic tree in Figure 1. Every column is a metabolic pathway. The darker the color the more complete the pathway, with the pale-yellow color indicating no detection and dark red indicating a complete pathway.

**Figure S4: Estimated genome completeness vs. subclade.** Estimated percent completion (percent) of SAR116 assembled genomes by subclade. Estimated genome completeness data were collected from CheckM output.

**Figure S5: Relative abundance of major SAR116 subclades by ecosystem from metagenomic recruitment of 1,059 metagenomes to all 349 SAR116 genomes**. Broad subclade classifications are ordered from the most abundant to least abundant subclade based on mean log10-transformed RPKM values. RPKM = reads per kilobase of genome per million mapped reads. Metagenomes were classified as Estuarine (A), Coastal (B), or Open Ocean (C). Pairwise comparisons of statistical significance between broad subclade classification means are represented in brackets above boxplots; “ns” = no significance, “****” = P value < 0.0001. D) World map showing the spatial distributions of the metagenomic samples used for recruitment to the SAR116 genomes. Every data point is a metagenomic sampling site, and marine environments are represented by color.

**Figure S6: Linear regression of log10-transformed subclade RPKM values vs. salinity values at all metagenomic sampling sites.** Data represented are from all three marine systems, and each panel represents a discrete SAR116 subclade. The trend line demonstrates the best fit from the quadratic regression model, and the shading around the trend line represents a 95% confidence interval of the fitted values. R-values quantify the correlation and directionality of the relationship between subclade RPKM and salinity.

**Figure S7: Linear regression of log10-transformed subclade RPKM values vs. salinity values at estuarine metagenomic sampling sites.** Data represented are from estuarine sites only, and each panel represents a discrete SAR116 subclade. The black trend line demonstrates the best fit from the quadratic regression model, and the shading around the trend line represents a 95% confidence interval of the fitted values. R-values quantify the correlation and directionality of the relationship between subclade RPKM and salinity.

**Figure S8: Linear regression of log10-transformed subclade RPKM values vs. salinity values at coastal metagenomic sampling sites.** Data represented are from coastal sites only, and each panel represents a discrete SAR116 subclade. The trend line demonstrates the best fit from the quadratic regression model, and the shading around the trend line represents a 95% confidence interval of the fitted values. R-values quantify the correlation and directionality of the relationship between subclade RPKM and salinity.

**Figure S9:** **Linear regression of log10-transformed subclade RPKM values vs. salinity values at open ocean metagenomic sampling sites.** Data represented are from open ocean sites only, and each panel represents a discrete SAR116 subclade. The trend line demonstrates the best fit from the quadratic regression model, and the shading around the trend line represents a 95% confidence interval of the fitted values. R-values quantify the correlation and directionality of the relationship between subclade RPKM and salinity.

**Figure S10: Linear regression of log10-transformed subclade RPKM values vs. temperature at all metagenomic sampling site.** Data represented are from all three marine systems, and each panel represents a discrete SAR116 subclade. The black trend line demonstrates the best fit from the quadratic regression model, and the shading around the trend line represents a 95% confidence interval of the fitted values. R-values quantify the correlation and directionality of the relationship between subclade RPKM and temperature.

**Figure S11: Linear regression of log10-transformed subclade RPKM values vs. temperature at coastal metagenomic sampling sites.** Data represented are from coastal sites only, and each panel represents a discrete SAR116 subclade. The black trend line demonstrates the best fit from the quadratic regression model, and the shading around the trend line represents a 95% confidence interval of the fitted values. R-values quantify the correlation and directionality of the relationship between subclade RPKM and temperature.

**Figure S12:** **Linear regression of log10-transformed subclade RPKM values vs. temperature at open ocean metagenomic sampling sites.** Data represented are from open ocean sites only, and each panel represents a discrete SAR116 subclade. The black trend line demonstrates the best fit from the quadratic regression model, and the shading around the trend line represents a 95% confidence interval of the fitted values. R-values quantify the correlation and directionality of the relationship between subclade RPKM and temperature.

**Figure S13: Quadratic regression of log10-transformed subclade RPKM values vs. temperature at estuarine metagenomic sampling sites.** Data represented are from estuarine sites only, and each panel represents a discrete SAR116 subclade. The black trend line demonstrates the best fit from the quadratic regression model, and the shading around the trend line represents a 95% confidence interval of the fitted values. R-values quantify the correlation and directionality of the relationship between subclade RPKM and temperature.

**Figure S14: Growth curves for SAR116 salinity tolerance.** Rows represent different salinity values in increasing order of salinity (ppt), and columns represent the different LSUCC isolates. Biological replicates are represented in each growth curve by different colors.

**Figure S15: Growth curves for SAR116 temperature tolerance.** Rows represent the different LSUCC isolates, and columns represent the different temperatures in increasing order of temperature (^o^C). Biological replicates are represented in each growth curve by different colors.

**Figure S16: Panel of SEM image size measurements for LSUCC0719.** Measurements taken are indicated by the red lines on each of the panels, and the corresponding measurement is recorded in red text next to the line.

**Figure S17: Panel of SEM image size measurements for LSUCC0744.** Measurements taken are indicated by the red lines on each of the panels, and the corresponding measurement is recorded in red text next to the line.

**Figure S18: Panel of SEM image size measurements for LSUCC0684.** Measurements taken are indicated by the red lines on each of the panels, and the corresponding measurement is recorded in red text next to the line.

**Figure S19:** **Phylogenetic tree of SoxB**. The scale bar represents 0.8 changes per position. S-sulfosulfanyl-L-cysteine sulfohydrolase, with closest relatives from NCBI RefSeq database, represented by black tips. Blue, green, orange, pink, and yellow tips indicate SAR116 SoxB protein sequences. Scale bar represents 0.08 changes per position. Bootstrap support values (n=1000) are indicated at nodes.

**Figure S20: Phylogenetic tree of FccB**. The scale bar represents 1 changes per position. Sulfide dehydrogenase [flavocytochrome c], with closest relatives from NCBI RefSeq database, represented by black tips. Blue, green, orange, pink, and yellow tips indicate SAR116 FccB protein sequences. Scale bar represents 1 change per position. Bootstrap support values (n=1000) are indicated at nodes.

**Figure S21:** **Phylogenetic tree of SoeA**. The scale bar represents 0.1 changes per position. Sulfite dehydrogenase (quinone), with closest relatives from NCBI RefSeq database, represented by black tips. Blue, green, purple and yellow tips indicate SAR116 SoeA protein sequences. Scale bar represents 0.1 changes per position. Bootstrap support values (n=1000) are indicated at nodes.

**Figure S21: Panel of LSUCC0684 (Calcibacteria**) **pleomorphy**. Measurements taken are indicated by the red lines on each of the panels, and the corresponding measurement is recorded in red text next to the line. LSUCC0684 “calcibacteria” are represented by the small flattened circular cells.

**Supplemental Tables**

**Table S1: Genome information.** Includes accession numbers for all SAR116 genomes and outgroup genomes. Along with the CheckM output for all SAR116 genomes, and a summary of genome completion by subclade.

**Table S2: Sulfur oxidation gene accession numbers.** Complete list of genomes and accession for genes included in figure 4. The data across the sheets in this table was manually selected from the Pangenome Summary (Table S5).

**Table S3: KEGGDecoder accessions** included in Figure 3. Along with the added accession numbers that we included for amino acid/trace metal/vitamin transport, and osmotic stress mitigation genes.

**Table S4: MWH2 medium recipe.**

**Table S5: Pairwise ANI vs. AAI matrix** of all SAR116 genomes. ANI values produced by FastANI and AAI values produced by CompareM.

**Table S6: Complete pangenome summary** of all SAR116 genomes from Anvi’o-7.1. Includes all gene annotations for all genomes, clustered in orthologous groups.

**Table S7:** **LSUCC SAR116 isolation data**. Includes the date the strain was isolated, along with the sampling location.

**Table S8: Environmental preference statistical results.** Statistical results for figures 5b-d, for open ocean, coastal, and estuarine systems. Kruskal-Wallis test determines if there are significant differences in RPKM values between the subclades. The Dunn test performs the pairwise test in mean RPKM values between subclades.

**Table S9: RPKM output with metadata.** Data for Figure 5. RPKM values from RRAP are summed by subclade. The location and depth of each sampling location is included per metagenome.

**Table S10: Cell measurements obtained via SEM images.** Data for Figure 7d-h. Includes raw measurements collected from ImageJ analysis, along with equations used to obtain final dimensions.

**References**

1. Henson, M.W., Pitre, D.M., Weckhorst, J.L., Lanclos, V.C., et al.: Artificial seawater media facilitate cultivating members of the microbial majority from the Gulf of Mexico. *mSphere*. 2016; 1(2):e00028-16. doi: 10.1128/mSphere.00028-16

2. Henson, M.W., Lanclos, V.C., Pitre, D.M., Weckhorst, J.L., et al.: Expanding the diversity of bacterioplankton isolates and modeling isolation eficacy with large-scale dilution-to-extinction cultivation. *Appl. Environ. Microbiol*. 2020; 86(17): e00943-20. doi: 10.1128/aem.00943-20

3. [Wick, R.: Porechop: adapter trimmer for Oxford Nanopore reads,](http://paperpile.com/b/rlUTZv/Vc7ks) <https://github.com/rrwick/Porechop>

4. [Bolger, A.M., Lohse, M., Usadel, B.: Trimmomatic: a flexible trimmer for Illumina sequence data. *Bioinformatics*. 2014; 30(15): 2114–2120. doi: 10.1093/bioinformatics/btu170](http://paperpile.com/b/rlUTZv/qEB9f)

5. [Wick, R.R., Judd, L.M., Gorrie, C.L., Holt, K.E.: Unicycler: Resolving bacterial genome assemblies from short and long sequencing reads. *PLoS Comput. Biol*. 2017; 13(6): e1005595. doi: 10.1371/journal/pcbi.1005595](http://paperpile.com/b/rlUTZv/ehHBV)

6. [Bankevich, A., Nurk, S., Antipov, D., Gurevich,et al.: SPAdes: a new genome assembly algorithm and its applications to single-cell sequencing. *J. Comput. Biol*. 2012; 19(5): 455–477. doi: 10.1089/cmb.2012.0021](http://paperpile.com/b/rlUTZv/nqZQP)

7. [Parks, D.H., Imelfort, M., Skennerton, C.T., Hugenholtz, P. et al.: CheckM: assessing the quality of microbial genomes recovered from isolates, single cells, and metagenomes. *Genome Res*. 2015; 25(7): 1043–1055. doi: 10.1101/gr.186072.114](http://paperpile.com/b/rlUTZv/dublE)

8. [Jain, C., Rodriguez-R, L.M., Phillippy, A.M., Konstantinidis, et al.: High throughput ANI analysis of 90K prokaryotic genomes reveals clear species boundaries. *Nat. Commun*. 2018; 9(5114). doi: 10.1038/s41467-018-07641-9](http://paperpile.com/b/rlUTZv/ZEz4C)

9. [Parks, D.H.: CompareM: A toolbox for comparative genomics,](http://paperpile.com/b/rlUTZv/tB7Kz) <https://github.com/donovan-h-parks/CompareM>

10. [Altschul, S.F., Gish, W., Miller, W., Myers, et al.: Basic local alignment search tool. *J. Mol. Biol*. 1990; 215(3): 403–410. doi: 10.1016/s0022-2836(05)80360-2](http://paperpile.com/b/rlUTZv/OsfHn)

11. [Konstantinidis, K.T., Tiedje, J.M.: Towards a genome-based taxonomy for prokaryotes. *J. Bacteriol*. 2005; 187(18); 6258–6264. doi: 10.1128/jb.187.186258-6264.2005](http://paperpile.com/b/rlUTZv/vLrl1)

12. [Konstantinidis, K.T., Rosselló-Móra, R., Amann, R.: Uncultivated microbes in need of their own taxonomy. *ISME J*. 2017; 11: 2399–2406. doi: 10.1038/ismej.2017.113](http://paperpile.com/b/rlUTZv/pOieo)

13. [Savoie, E.R., Lanclos, V.C., Henson, M.W., Cheng, C., et al.: Ecophysiology of the cosmopolitan OM252 bacterioplankton (*Gammaproteobacteria*). *mSystems*. 2021; 6(3): e0027621. doi: 10.1128/msystems.00276-21](http://paperpile.com/b/rlUTZv/zx8cJ)

14. [Lanclos, V.C., Rasmussen, A.N., Kojima, C.Y., Cheng, C., et al.: Ecophysiology and genomics of the brackish water adapted SAR11 subclade IIIa. *ISME J*. 2023; 17(4): 620–629. doi: 10.1038/s41396-023-01376-2](http://paperpile.com/b/rlUTZv/4Snlv)

15. [Kojima, C.Y., Getz, E.W., Thrash, J.C.: RRAP: RPKM recruitment analysis pipeline. *Microbiol Resour Announc*. 2022; 11(9): e0064422. doi: 10/1128/mra.00644-22](http://paperpile.com/b/rlUTZv/3fFjN)

16. [Kruskal, W.H., Wallis, W.A.: Use of ranks in one-Cciterion variance analysis. *J. Am. Stat. Assoc*. 1952; 47(260): 583–621. doi: 10.1080/01621459.1952.10483441](http://paperpile.com/b/rlUTZv/hn56N)

17. [Dunn, O.J.: Multiple comparisons among means. *J. Am. Stat. Assoc*. 1961; 56(293): 52–64. doi: 10.2307/2282330](http://paperpile.com/b/rlUTZv/wo73o)

18. [R: A language and environment for statistical computing,](http://paperpile.com/b/H8ueyx/sqlIe) [https://www.R-project.org/](https://www.r-project.org/)

19. Posit team (2025). [RStudio: Integrated Development for R.](http://paperpile.com/b/H8ueyx/6mkYU) Posit Software, PBC, Boston, MA. *http://www.posit.co/*

20. [Ahmed, M.A., Lim, S.J., Campbell, B.J.: Metagenomes, metatranscriptomes, and metagenome-assembled genomes from chesapeake and delaware bay (USA)water samples. *Microbiol Resour Announc*. 2021; 10(21), e0026221. doi: 10.11128/mra.00262-21](http://paperpile.com/b/H8ueyx/qCSH)

21. [Damashek, J., Edwardson, C.F., Tolar, B.B., Gifford, S.M., et al.: Coastal ocean metagenomes and curated metagenome-assembled genomes from Marsh Landing, Sapelo Island (Georgia, USA). *Microbiol Resour Announc*. 2019; 8(40): e00934-19. doi: 10.1128.mra.00934-19](http://paperpile.com/b/H8ueyx/G6LZ)

22. [Xu, B., Li, F., Cai, L., Zhang, R., et al.: A holistic genome dataset of bacteria, archaea and viruses of the Pearl River estuary. *Sci Data*. 2022; 9(1): 49. doi: 10.1038/s41597-022-01153-4](http://paperpile.com/b/H8ueyx/8ACl)

23. [Sieradzki, E.T., Morando, M., Fuhrman, J.A.: Metagenomics and quantitative stable isotope probing offer insights into metabolism of polycyclic aromatic hydrocarbon degraders in chronically polluted seawater. *mSystems*. 2021; 6(3): e00245-2. doi: 10.1128/msystems.00245-21](http://paperpile.com/b/H8ueyx/kuYK)

24. [Lee, J., Kwon, K.K., Lim, S.-I., Song, J., et al.: Isolation, cultivation, and genome analysis of proteorhodopsin-containing SAR116-clade strain Candidatus *Puniceispirillum marinum* IMCC1322. *J. Microbiol*. 2019; 57(8): 676–687. doi: 10.1007/s12275-019-9001-2](http://paperpile.com/b/rlUTZv/yP2AZ)

25. [Bonthond, G., Merselis, D.G., Dougan, K.E., Graff, T., et al.: Inter-domain microbial diversity within the coral holobiont *Siderastrea siderea* from two depth habitats. *PeerJ*. 2018; 6: e4323. doi: 10.7717/peerj.4323](http://paperpile.com/b/rlUTZv/bd4HX)

26. [Quast, C., Pruesse, E., Yilmaz, P., Gerken, J., et al.: The SILVA ribosomal RNA gene database project: improved data processing and web-based tools. *Nucleic Acids Res*. 2013; 41(D1) D590–D596. doi: 10.1093/nar/gks1219](http://paperpile.com/b/rlUTZv/koqIb)

27. [Yilmaz, P., Yarza, P., Rapp, J.Z., Glöckner, F.O.: Expanding the world of marine bacterial and archaeal clades. *Front. Microbiol*. 2015; 6: 1524. doi: 10/3389/fmicb.2015.01524](http://paperpile.com/b/rlUTZv/zFKR1)

28. Beccati, A., Gerken, J., Quast, C., Yilmaz, P., et al.: SILVA tree viewer: interactive web browsing of the SILVA phylogenetic guide trees. BMC Bioinformatics. 2017; 18(1): 433. doi: 10.1186/s12859-017-1841-3

29. [Capella-Gutiérrez, S., Silla-Martínez, J.M., Gabaldón, T.: trimAl: a tool for automated alignment trimming in large-scale phylogenetic analyses. *Bioinformatics*. 2009; 25(15): 1972–1973. doi: 10.1093/bioinformatics/btp348](http://paperpile.com/b/rlUTZv/wKG9q)

30. [Edgar, R.C.: MUSCLE: multiple sequence alignment with high accuracy and high throughput. *Nucleic Acids Res*. 2004; 32(5): 1792–1797. doi: 10.1093/nar/gkh340](http://paperpile.com/b/rlUTZv/00pX5)

31. [Nguyen, L.-T., Schmidt, H.A., von Haeseler, A., Minh, B.Q.: IQ-TREE: a fast and effective stochastic algorithm for estimating maximum-likelihood phylogenies. *Mol. Biol. Evol*. 2015; 32(1), 268–274. doi: 10.1093/molbev/msu300](http://paperpile.com/b/rlUTZv/ShGrl)

32. [Yu, G.: Using ggtree to Visualize Data on Tree-Like Structures. *Curr. Protoc. Bioinformatics*. 2020; 69(1), e96. doi: 10.1002/cpbi.96](http://paperpile.com/b/rlUTZv/1UyRS)

33. [Yu, G., Lam, T.T.-Y., Zhu, H., Guan, Y.: Two methods for mapping and visualizing associated data on phylogeny using Ggtree. *Mol. Biol. Evol*. 2018; 35(12): 3041–3043. doi: 10.1039/molbev/msy194](http://paperpile.com/b/rlUTZv/TBJQv)

34. [Yu, G., Smith, D.K., Zhu, H., Guan, Y., et al.: Ggtree: An r package for visualization and annotation of phylogenetic trees with their covariates and other associated data. *Methods Ecol. Evol*. 2017; 8: 28–36. doi: 10.1111/20241-210x.12628](http://paperpile.com/b/rlUTZv/XmkLd)

35. [Frigaard, N.-U., Dahl, C.: Sulfur metabolism in phototrophic sulfur bacteria. *Adv. Microb. Physiol*. 2009; 54: 103–200. doi: 10.1016/s0065-2911(09)00002-7](http://paperpile.com/b/rlUTZv/znW2z)

36. [Dahl, C., Franz, B., Hensen, D., Kesselheim, A., et al.: Sulfite oxidation in the purple sulfur bacterium Allochromatium vinosum: identification of SoeABC as a major player and relevance of SoxYZ in the process. *Microbiology*. 2013; 159(Pt 12): 2626–2638. doi: 10.1099/mic.0.071019-0](http://paperpile.com/b/H8ueyx/0H3De)

37. [O’Leary, N.A., Wright, M.W., Brister, J.R., Ciufo, S., et al.: Reference sequence (RefSeq) database at NCBI: current status, taxonomic expansion, and functional annotation. *Nucleic Acids Res*. 2016; 44(D1): D733–D745. doi: 10.1093/nar/gkv1189](http://paperpile.com/b/rlUTZv/kfzPY)

38. [Lewis, E.L.: The practical salinity scale of 1978 and its antecedents. *IEEE J. Ocean. Eng.* Geod. 1980; 5(1): 3-8. doi: 10.1109/JOE.1980.1145448](http://paperpile.com/b/H8ueyx/aIMXw)

39. [Cheng, C., Thrash, J.C.: sparse-growth-curve: a computational pipeline for parsing cellular growth curves with low temporal resolution. *Microbiol Resour Announc*. 2021; 10(19): e00296. doi: 10.1128/mra.00296-21](http://paperpile.com/b/rlUTZv/CNJGB)

40. Wickham H: ggplot2: Elegant graphics for data analysis. Springer Verlag New York. ISBN 978-3-319-242774. <https://ggplot2.tidyverse.org>

41. [Schneider, C.A., Rasband, W.S., Eliceiri, K.W.: NIH Image to ImageJ: 25 years of image analysis. *Nat. Methods*. 2012; 9(7); 671–675. doi: 10.1038/nmeth.2089](http://paperpile.com/b/rlUTZv/sN7ra)

42. [Bartlett, M.S., Fowler, R.H.: Properties of sufficiency and statistical tests. *Proc R. Soc. Lon. Series A - Mathematical and Physical Sciences*. 1997; 160(901): 268–282. doi: 10.1098/rspa.1937.0109](http://paperpile.com/b/H8ueyx/zMaNa)

43. [Welch, B.L.: On the Comparison of Several Mean Values: An Alternative Approach. *Biometrika*. 1951; 38(3/4) 330–336. doi: 10.2307/2332579](http://paperpile.com/b/H8ueyx/mqZfi)

44. [Fisher, R.A.: Statistical methods for research workers. In: Springer Series in Statistics. pp. 66–70. Springer New York, New York, NY (1992)](http://paperpile.com/b/H8ueyx/b1Exz)

45. [Kassambara, A.: rstatix: Pipe-Friendly Framework for Basic Statistical Tests. (2023)](http://paperpile.com/b/H8ueyx/SdGh9) <https://rpkgs.datanovia.com/rstatix/>

46. [Green, G.N., Lorence, R.M., Gennis, R.B.: Specific overproduction and purification of the cytochrome b558 component of the cytochrome d complex from Escherichia coli. *Biochemistry*. 1986; 25(9): 2309–2314. doi: 10.1021/bi00357a002](http://paperpile.com/b/H8ueyx/ANHu7)

47. [Guo, R., Ma, X., Zhang, J., Liu, C., et al.: Microbial community structures and important taxa across oxygen gradients in the Andaman Sea and eastern Bay of Bengal epipelagic waters. *Front. Microbiol*. 2022; 13: 1041521. doi: 10.3389/fmicb.2022.1041521](http://paperpile.com/b/H8ueyx/fjOyR)

48. [Dias, B., Weimer, B.: Conversion of methionine to thiols by lactococci, lactobacilli, and brevibacteria. *Appl. Environ. Microbiol*. 1998; 64(9): 3320–3326. doi: 10.1128/aem.64.9.3320-3326.1998](http://paperpile.com/b/H8ueyx/ahudf)

49. [Miller, A.R., North, J.A., Wildenthal, J.A., Tabita, F.R.: Two Distinct Aerobic Methionine Salvage Pathways Generate Volatile Methanethiol in Rhodopseudomonas palustris. *MBio*. 2018; 9(2) . doi: 10.1128/mbio.00407-18](http://paperpile.com/b/H8ueyx/FZQfR)

50. [Kitabatake, M., So, M.W., Tumbula, D.L., Söll, D.: Cysteine biosynthesis pathway in the archaeon Methanosarcina barkeri encoded by acquired bacterial genes? *J. Bacteriol*. 2000; 182(1): 143–145. doi: 10.1128/jb.182.1.143-145.2000](http://paperpile.com/b/H8ueyx/sgtKP)

51. [He, Y., Zeng, X., Xu, F., Shao, Z.: Diversity of Mixotrophic Neutrophilic Thiosulfate- and Iron-Oxidizing Bacteria from Deep-Sea Hydrothermal Vents. *Microorganisms*. 2022; 11(1): 100. doi: 10.3390/microorganisms11010100](http://paperpile.com/b/rlUTZv/zzWWB)

52. [Trivedi, C.B., Stamps, B.W., Lau, G.E., Grasby, et al.: Microbial Metabolic Redundancy Is a Key Mechanism in a Sulfur-Rich Glacial Ecosystem. *mSystems*. 2020; 5(4): e00504-20. doi: 10.1128/msystems.00504-20](http://paperpile.com/b/rlUTZv/g4qW3)

53. [Dalcin Martins, P., Echeveste Medrano, M.J., Arshad, A., Kurth, et al.: Unraveling Nitrogen, Sulfur, and Carbon Metabolic Pathways and Microbial Community Transcriptional Responses to Substrate Deprivation and Toxicity Stresses in a Bioreactor Mimicking Anoxic Brackish Coastal Sediment Conditions. *Front. Microbiol*. 2022; 13: 798906. doi: 10.3389/fmicb.2022.798906](http://paperpile.com/b/rlUTZv/WyF4t)

54. [Thomas, F., Giblin, A.E., Cardon, Z.G., Sievert, S.M.: Rhizosphere heterogeneity shapes abundance and activity of sulfur-oxidizing bacteria in vegetated salt marsh sediments. *Front. Microbiol*. 2014; 5: 309. doi: 10.3389/fmicb.2014.00309](http://paperpile.com/b/rlUTZv/HL1qf)

55. [Luo, J., Tan, X., Liu, K., Lin, W.: Survey of sulfur-oxidizing bacterial community in the Pearl River water using *soxB*, *sqr*, and *dsrA* as molecular biomarkers. *3 Biotech*. 2018; 8(1): 73. doi: 10.1007/s13205-017-1077-y](http://paperpile.com/b/rlUTZv/307wm)
