# Supplementary material for: Culture-supported ecophysiology of the SAR116 clade demonstrates metabolic and spatial niche partitioning
Source: ISME J. 2025 Jun 13;19(1):wraf124. doi: 10.1093/ismejo/wraf124 (PMC12262181; doi:10.1093/ismejo/wraf124)
Supplement: supplementary-material_wraf124 [file supplementary-material_wraf124.zip › FigureS2_16S_tree_wraf124.pdf]

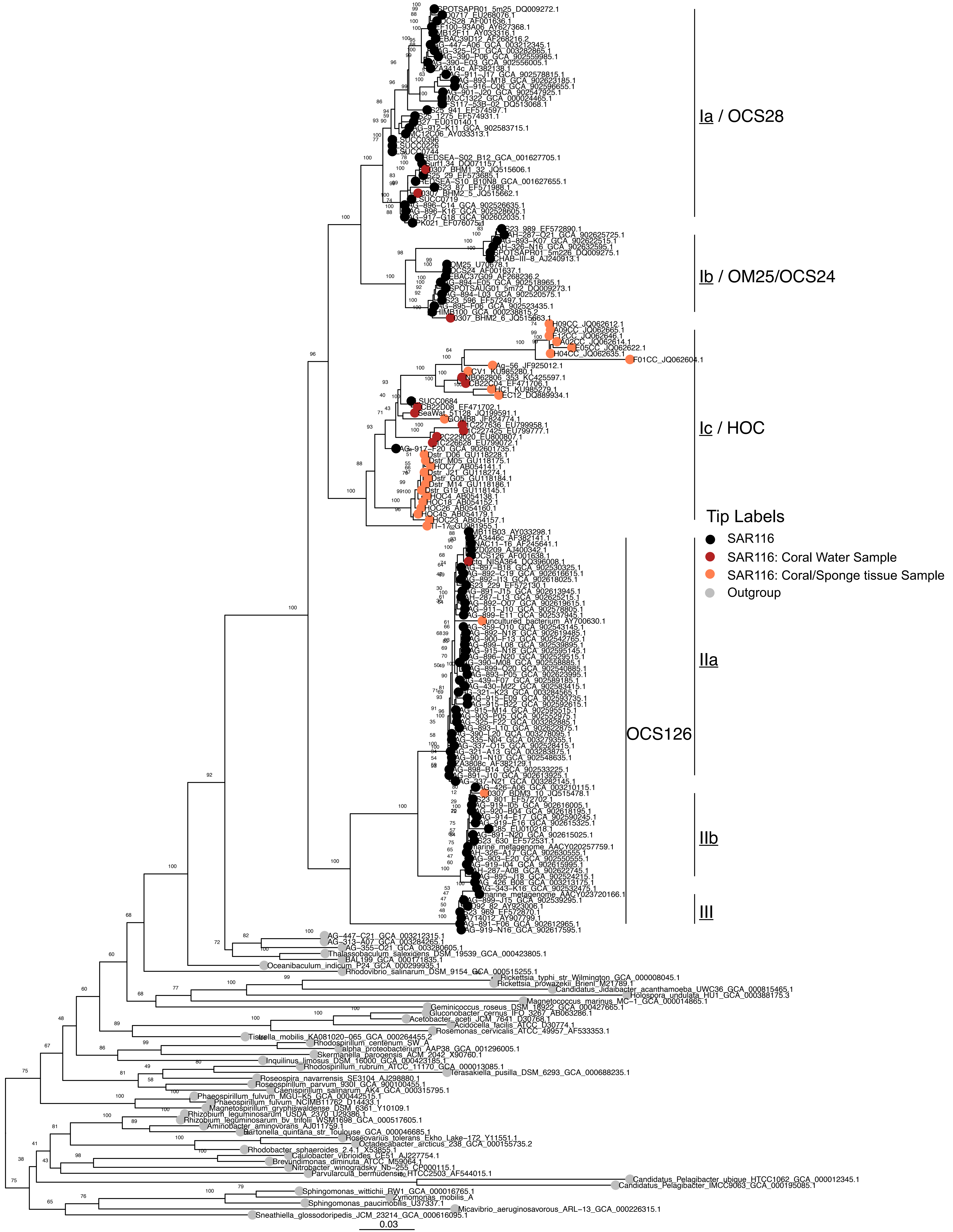
Ia / OCS28
Ib / OM25/OCS24
Ic / HOC
Tip Labels
SAR116
SAR116: Coral Water Sample
SAR116: Coral/Sponge tissue Sample
Outgroup
IIa
OCS126
IIb
III
0.03
