# Supplementary figures and images for: Culture-supported ecophysiology of the SAR116 clade demonstrates metabolic and spatial niche partitioning
Source: ISME J. 2025 Jun 13;19(1):wraf124. doi: 10.1093/ismejo/wraf124 (PMC12262181; doi:10.1093/ismejo/wraf124)

# Linear regression of temperature vs Subclade RPKM

Open ocean, coastal, and estuarine systems

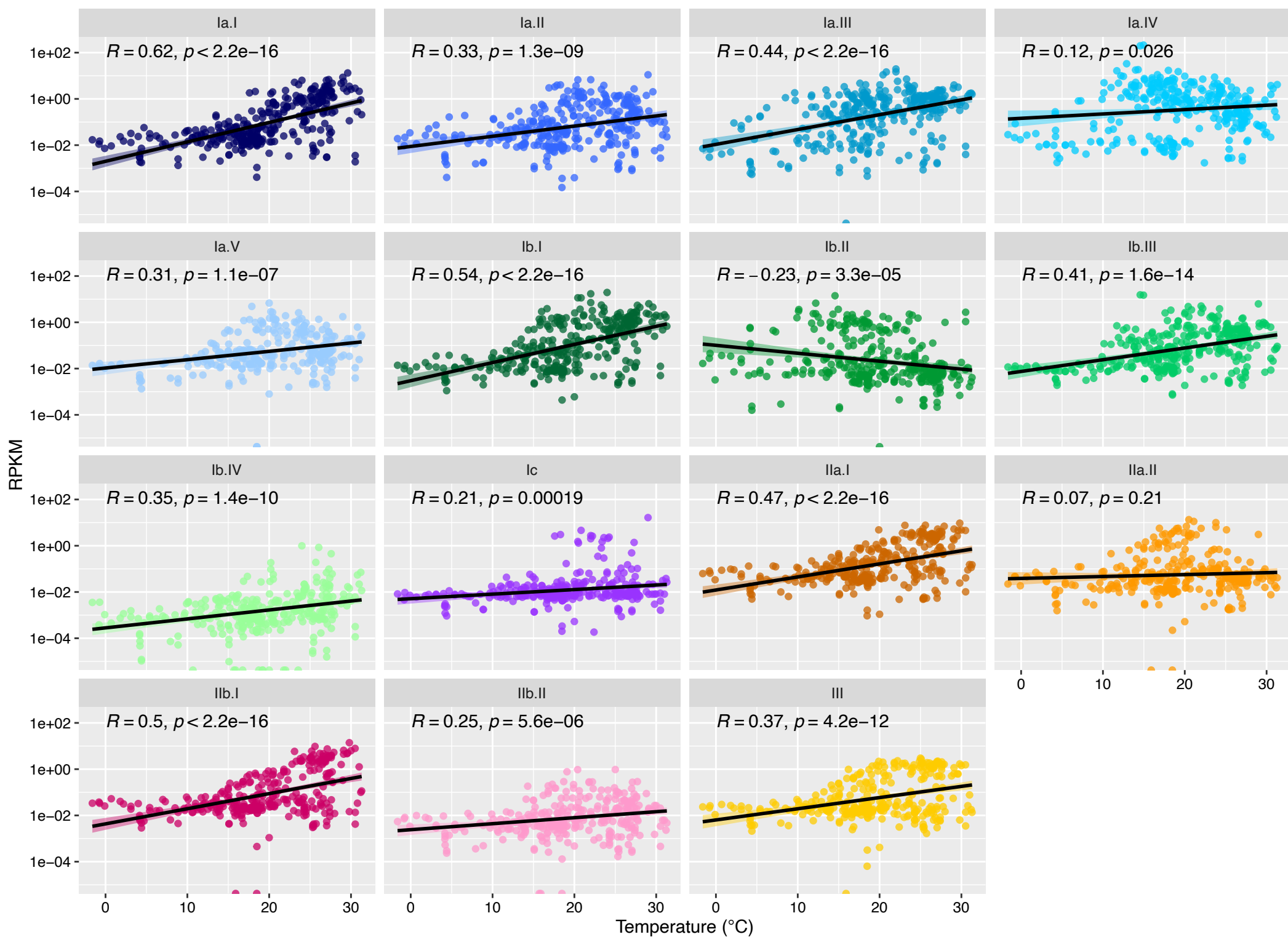

Supplement: supplementary-material_wraf124 [file supplementary-material_wraf124.zip › FigureS10_LinearRegression_Temperature_wraf124.pdf]

# Linear Regression of temperature vs Subclade RPKM

Coastal systems

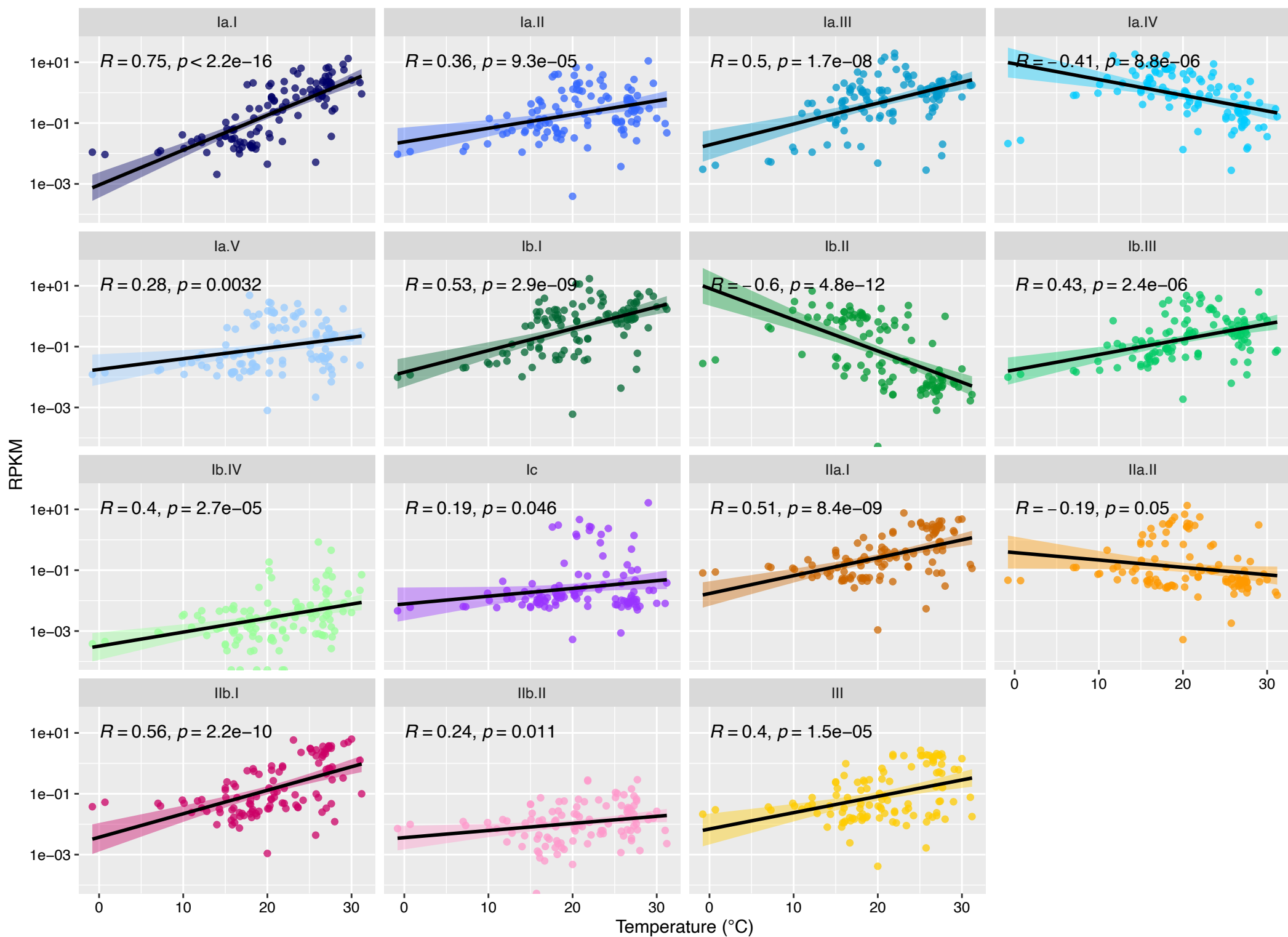

Supplement: supplementary-material_wraf124 [file supplementary-material_wraf124.zip › FigureS11_LinearRegression_Temp_Coastal_wraf124.pdf]

# Linear regression of temperature vs Subclade RPKM

Open ocean systems

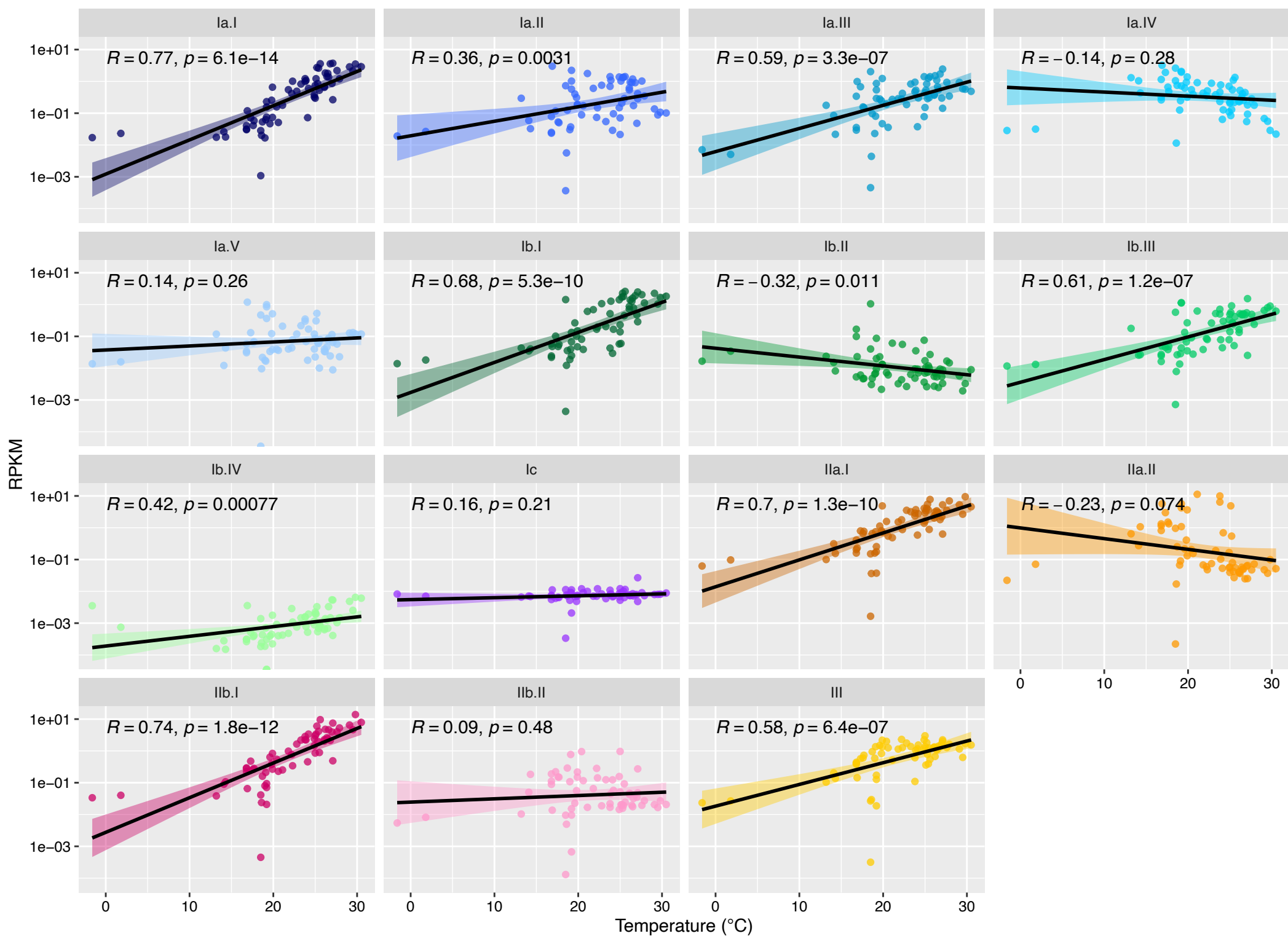

Supplement: supplementary-material_wraf124 [file supplementary-material_wraf124.zip › FigureS12_LinearRegression_Temp_OpenOcean_wraf124.pdf]

# Linear regression of temperature vs Subclade RPKM

Estuarine systems

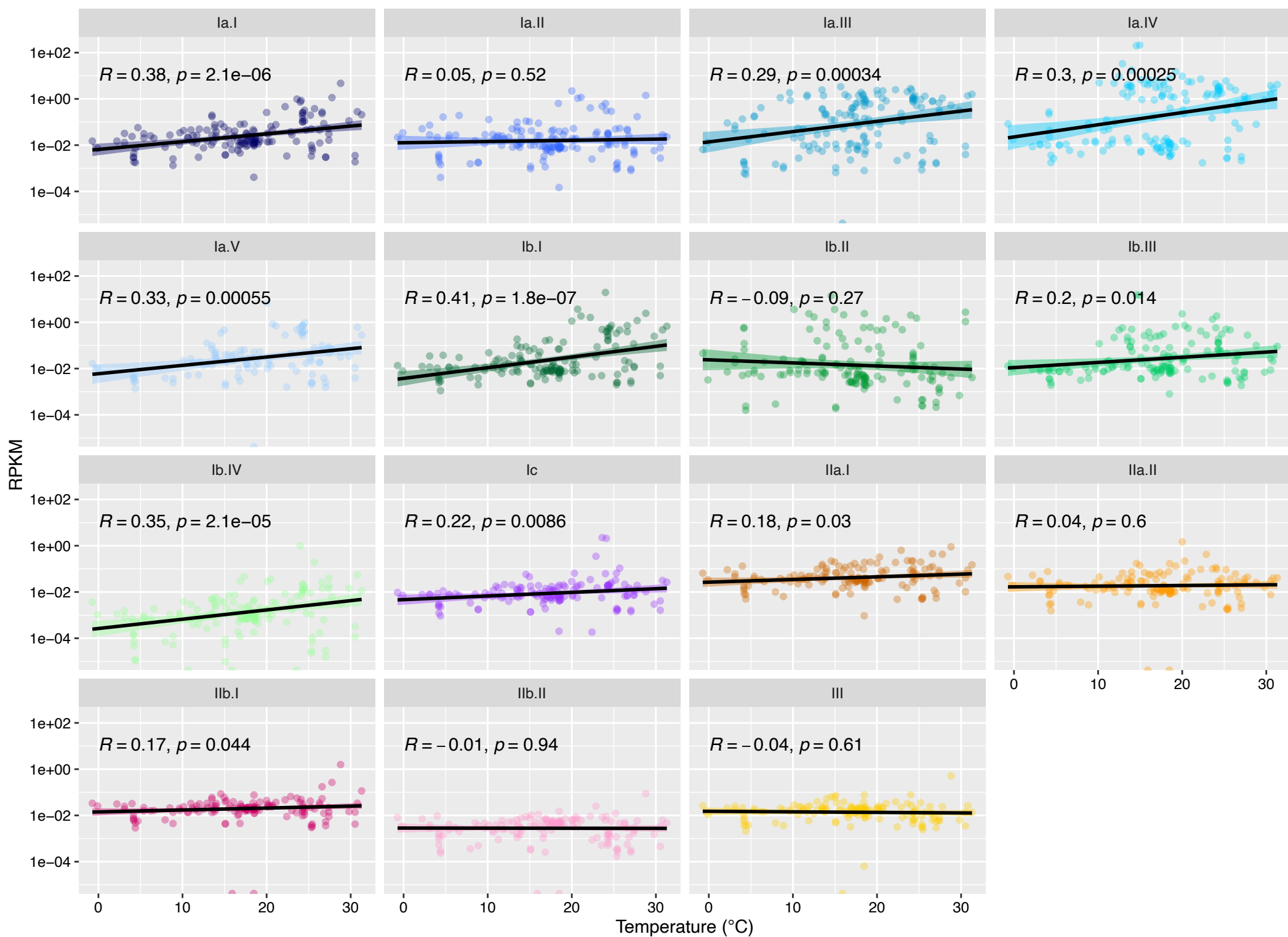

Supplement: supplementary-material_wraf124 [file supplementary-material_wraf124.zip › FigureS13_LinearRegression_Temp_Estuarine_wraf124.pdf]

# SAR116 salinity growth tolerance

Cell density vs. Time across 5 salinity values

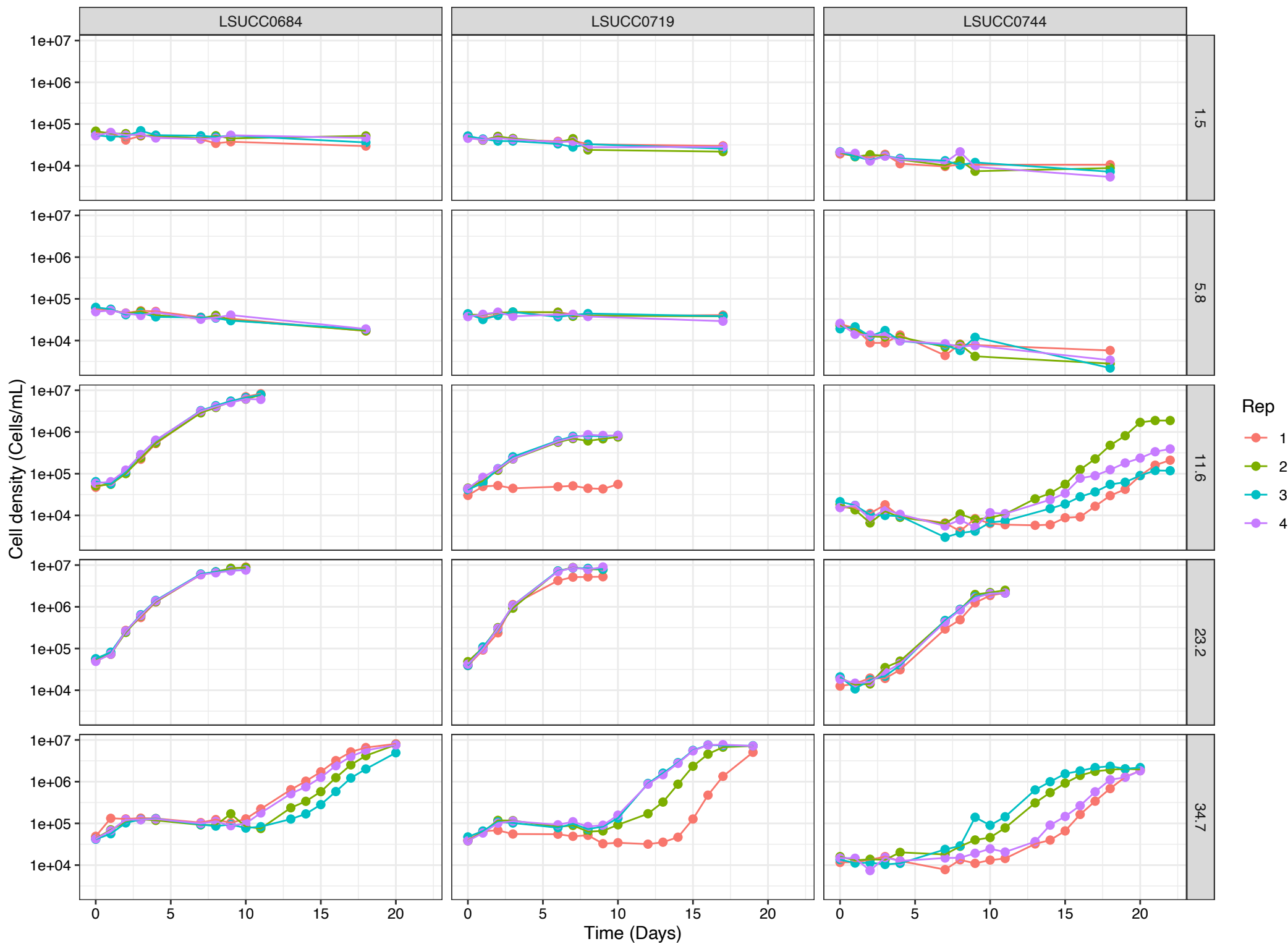

Supplement: supplementary-material_wraf124 [file supplementary-material_wraf124.zip › FigureS14_684_719_744_SalinityExp_wraf124.pdf]

# SAR116 Temperature Growth Tolerance

Cell Density vs. Time across 7 temperatures

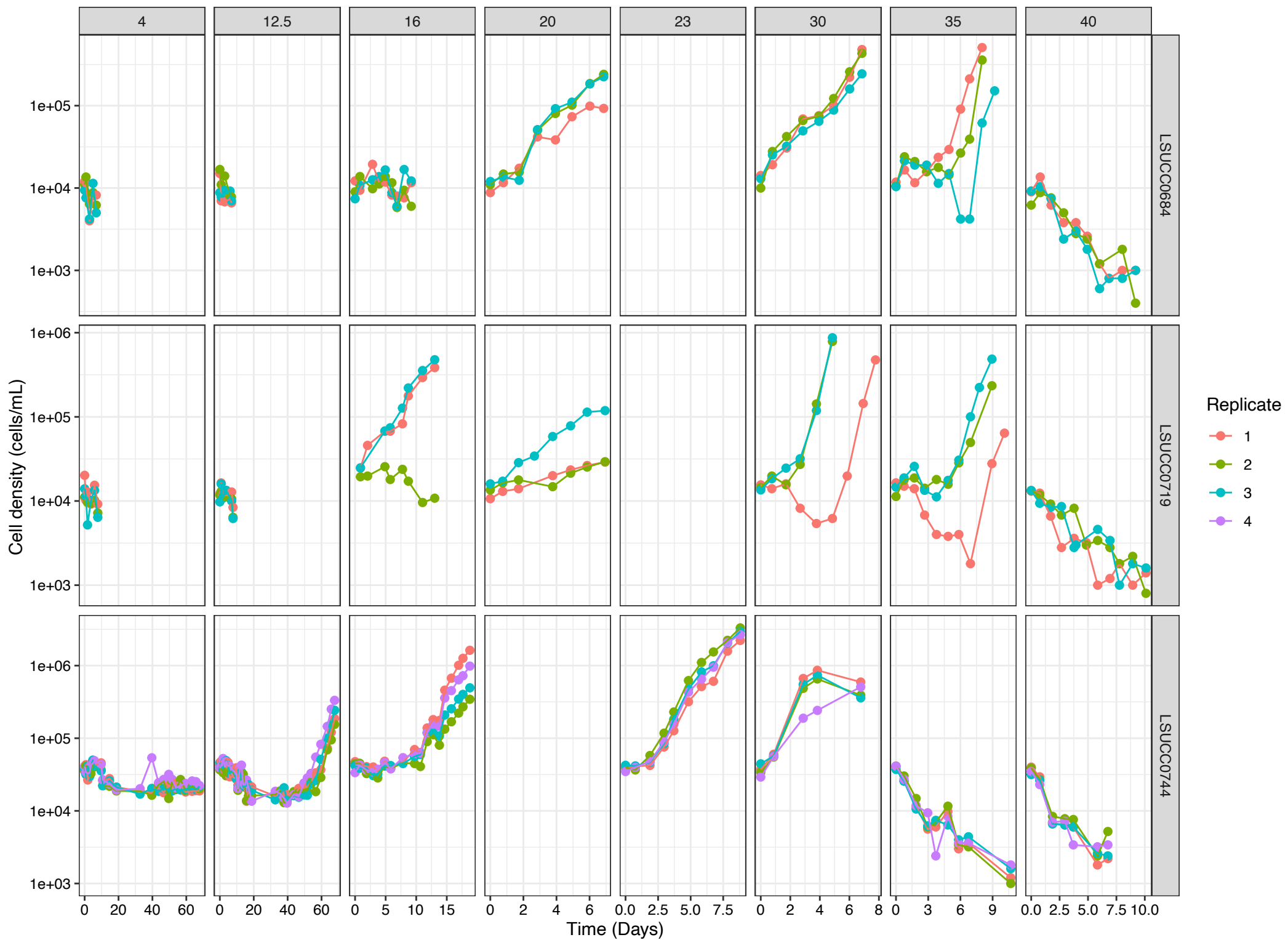

Supplement: supplementary-material_wraf124 [file supplementary-material_wraf124.zip › FigureS15_684_719_744_TempExp_wraf124.pdf]

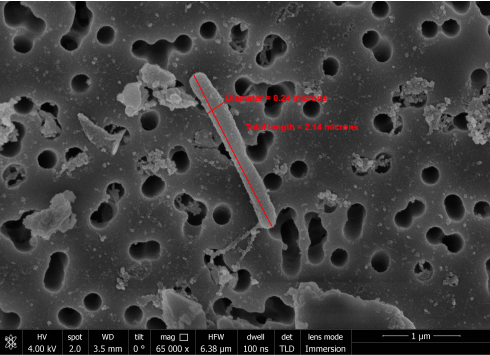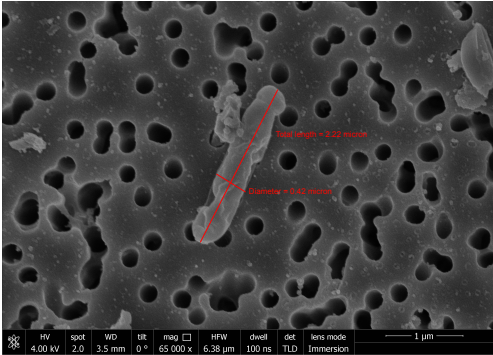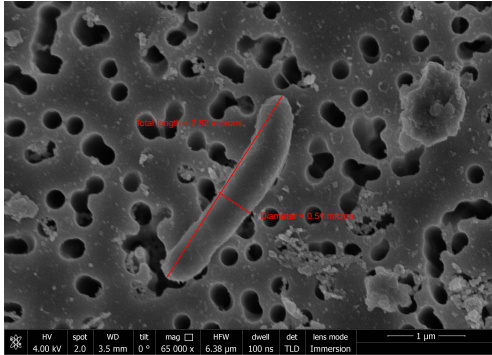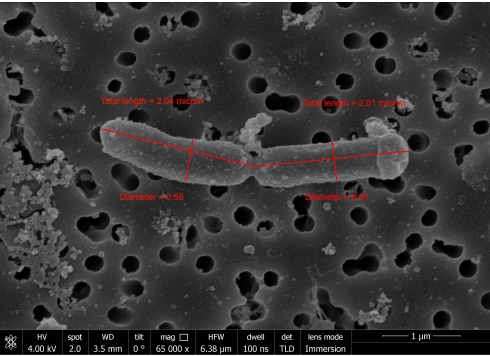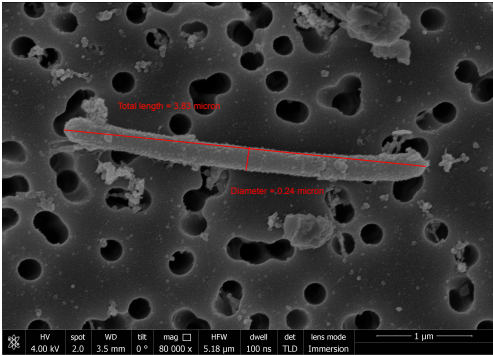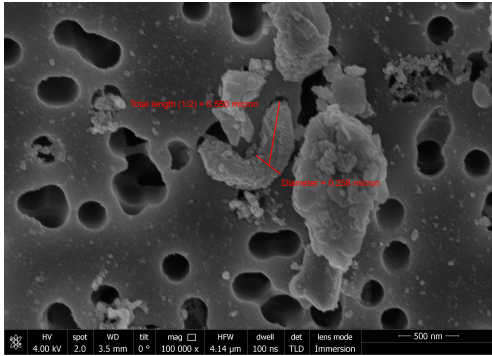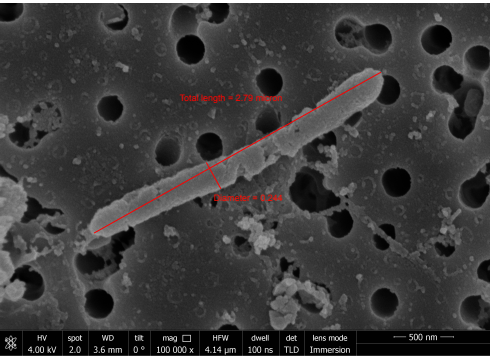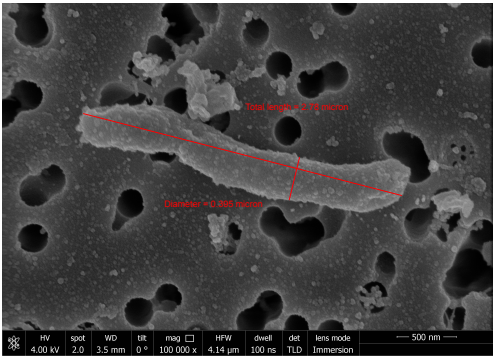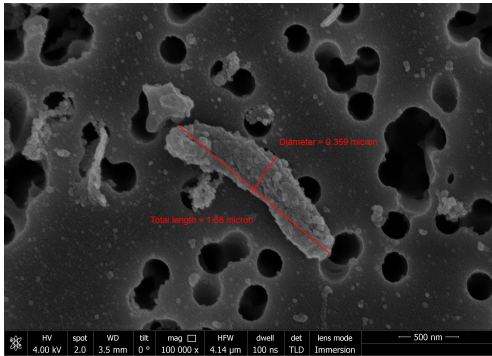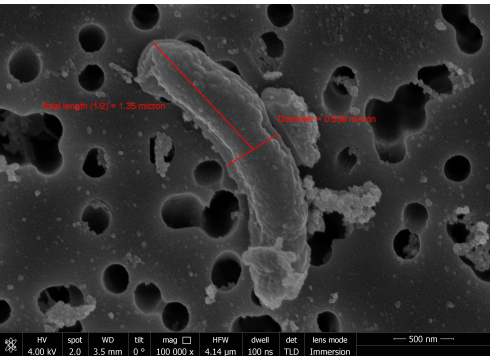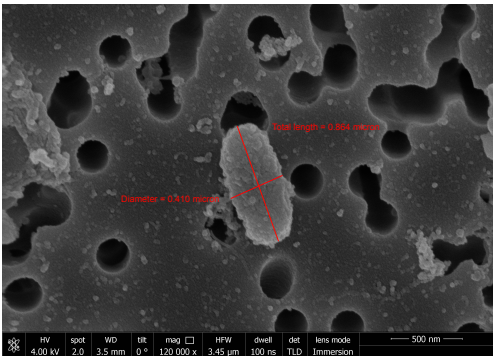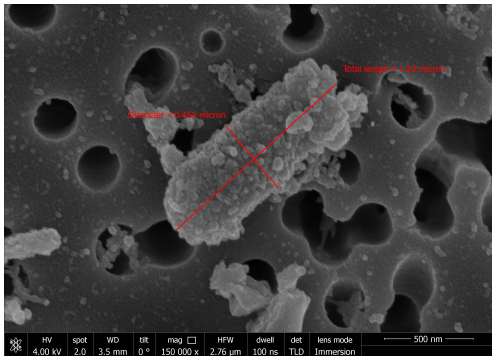

Supplement: supplementary-material_wraf124 [file supplementary-material_wraf124.zip › FigureS16_LSUCC0719_size_panel_wraf124.pdf]

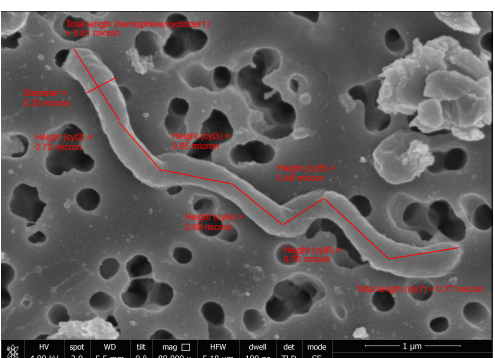

Supplement: supplementary-material_wraf124 [file supplementary-material_wraf124.zip › FigureS18_LSUCC0684_size_panel_wraf124.pdf]

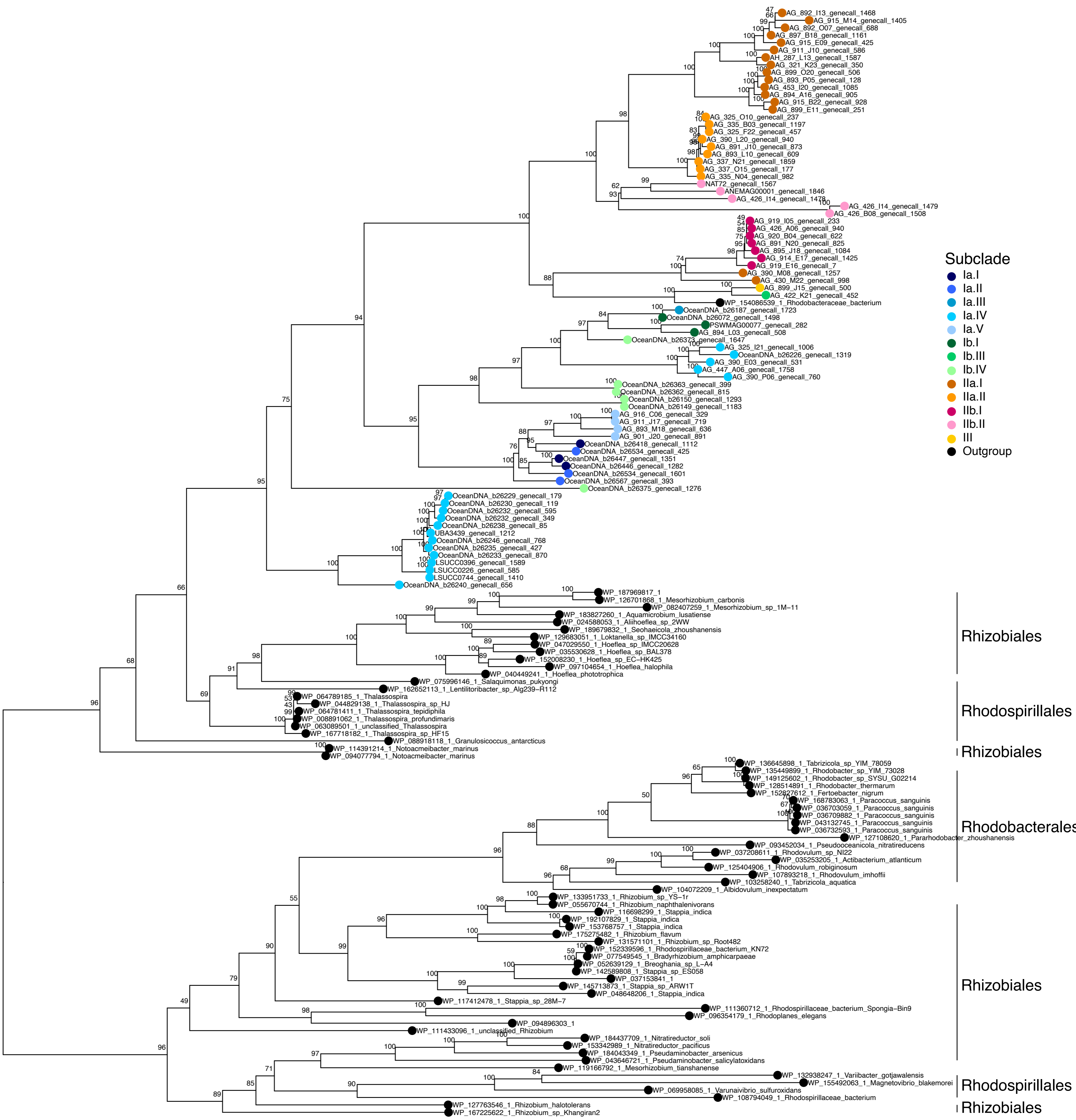

Supplement: supplementary-material_wraf124 [file supplementary-material_wraf124.zip › FigureS19_SoxB_tree_wraf124.pdf]

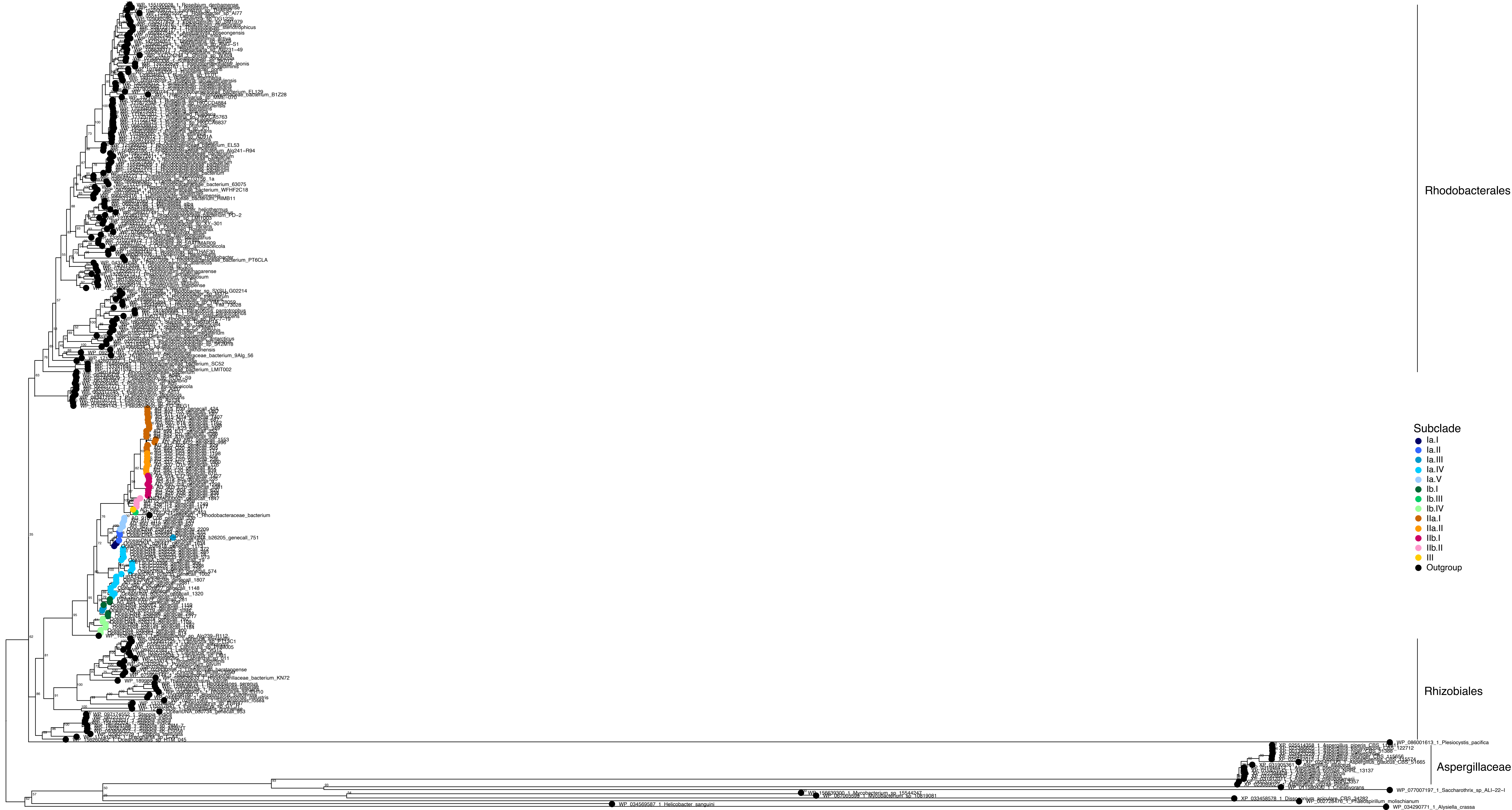

Supplement: supplementary-material_wraf124 [file supplementary-material_wraf124.zip › FigureS20_FccB_tree_wraf124.pdf]

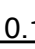

Supplement: supplementary-material_wraf124 [file supplementary-material_wraf124.zip › FigureS21_soeA_ggtree_wraf124.pdf]

A

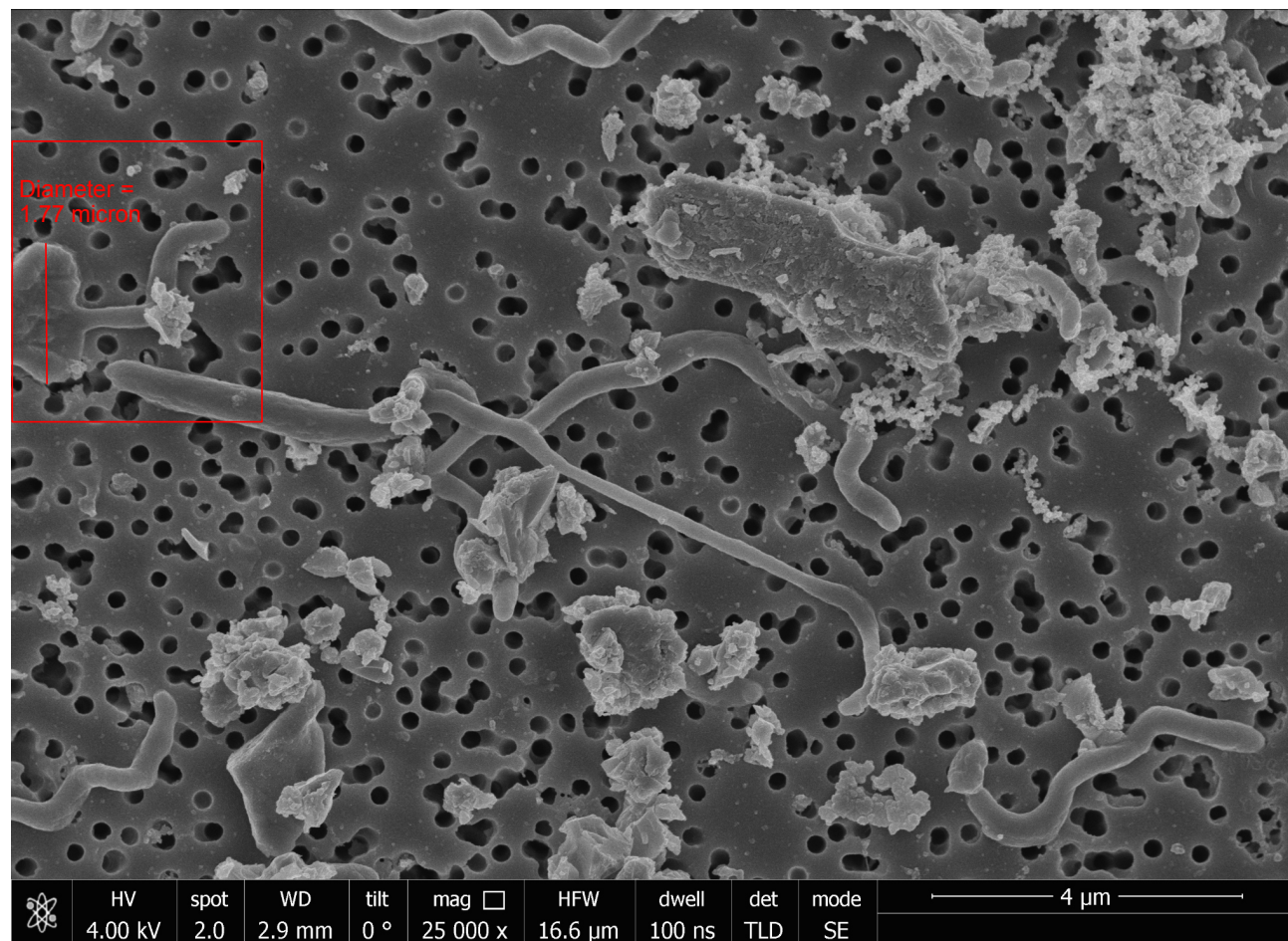

B

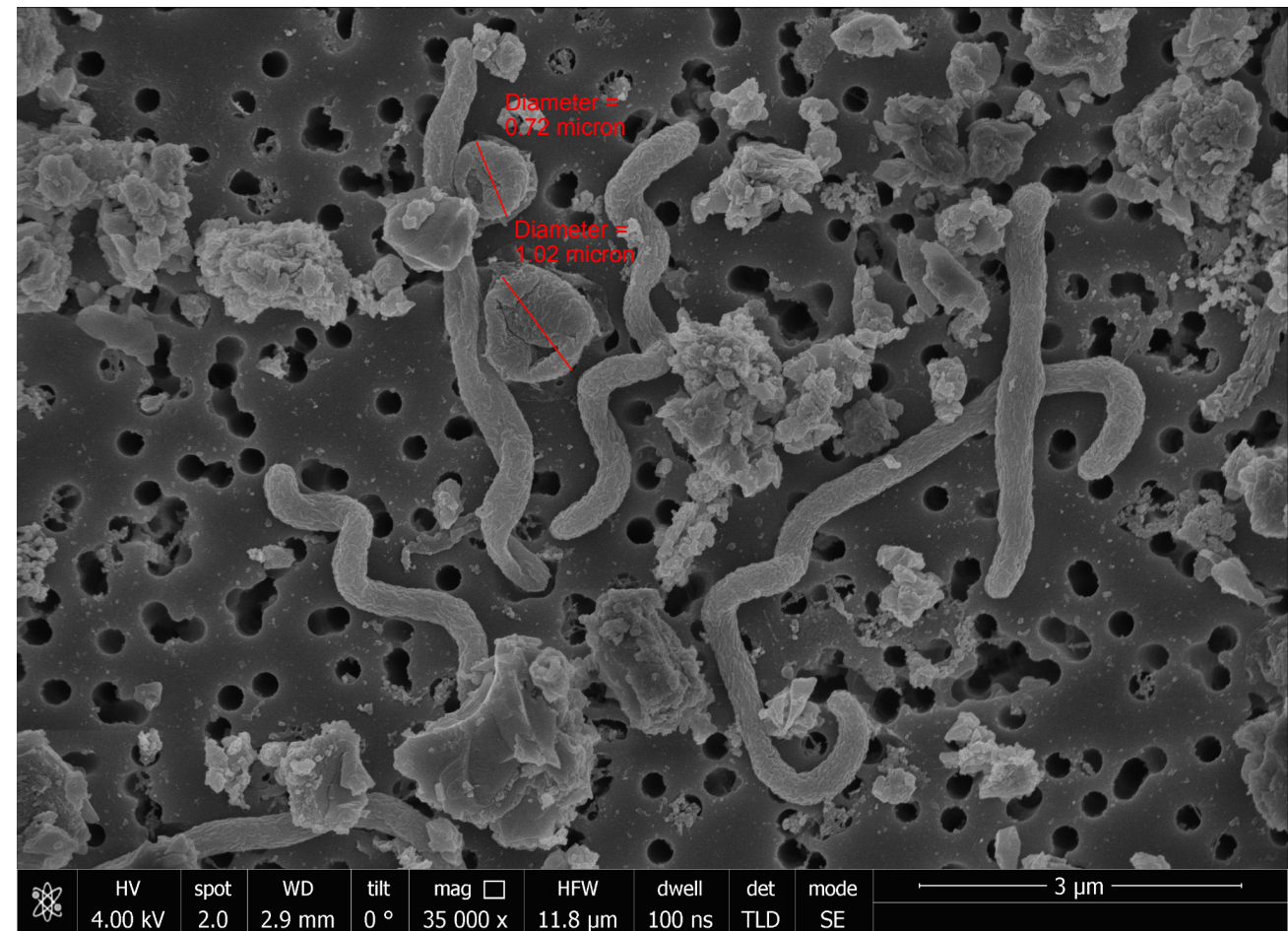

C

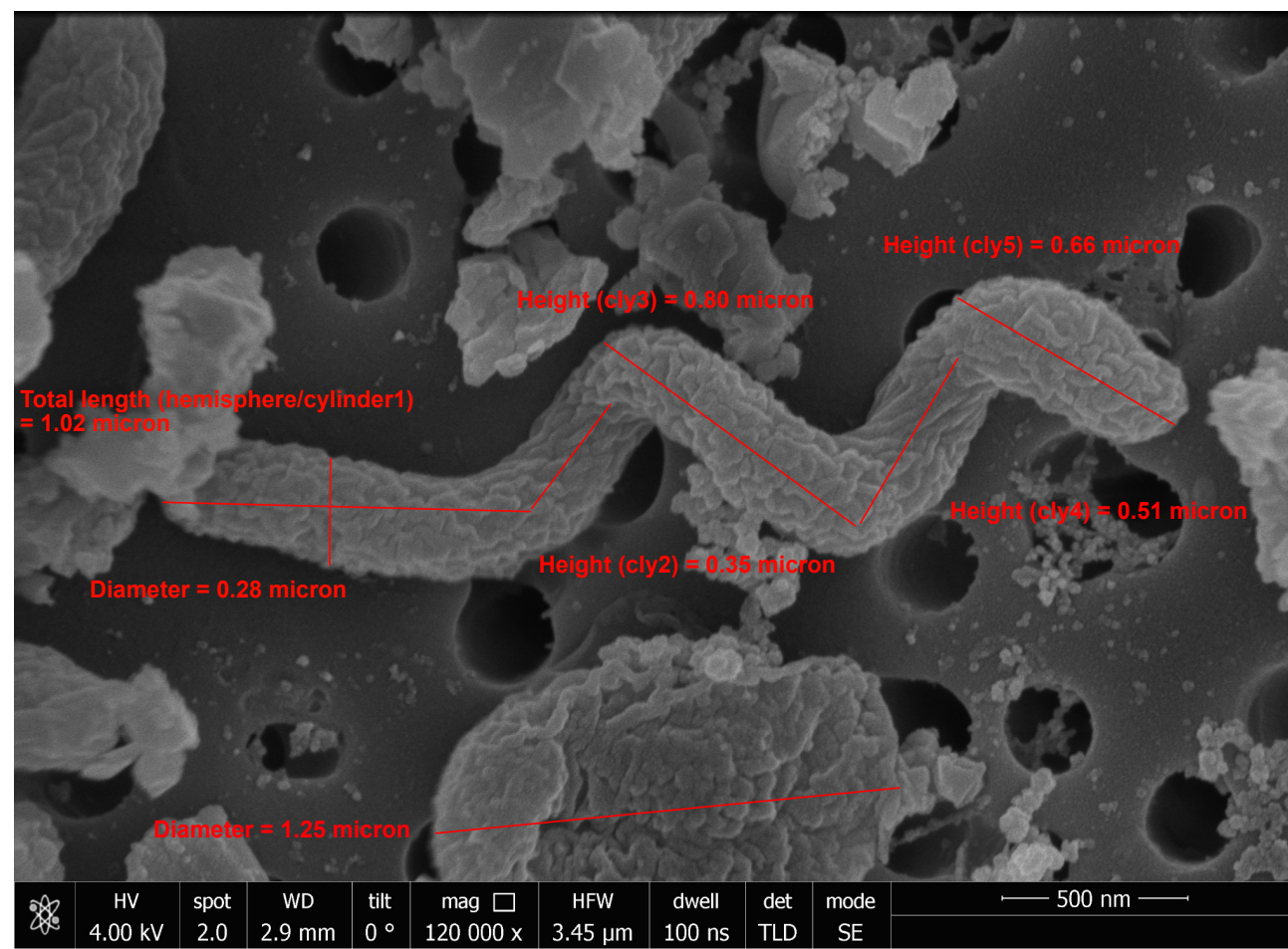

D

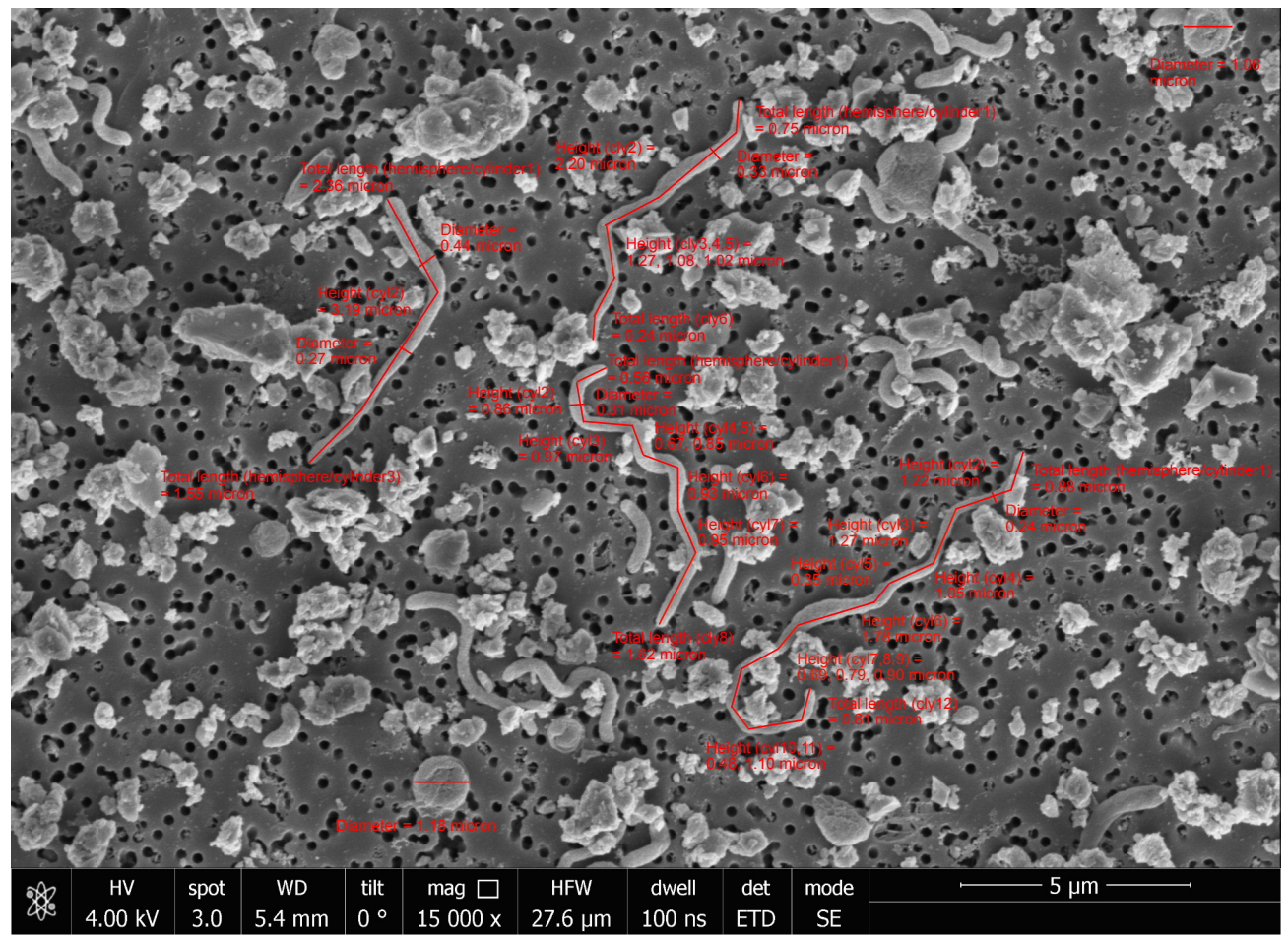

Supplement: supplementary-material_wraf124 [file supplementary-material_wraf124.zip › FigureS22_Calcibacteria_life_cycle_wraf124.pdf]

# Estimated genome completeness vs. Subclade

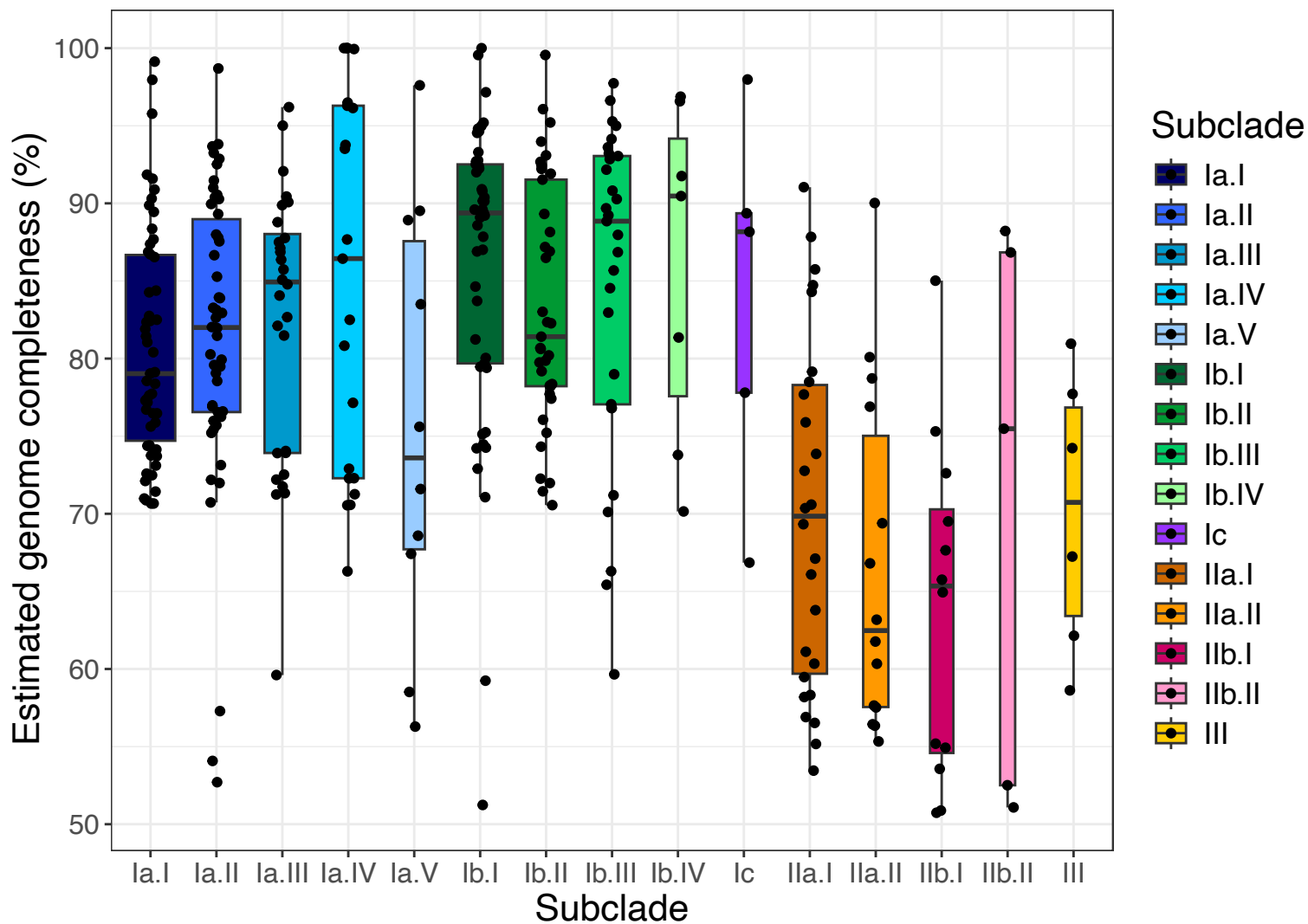

Supplement: supplementary-material_wraf124 [file supplementary-material_wraf124.zip › FigureS4_GenomeCompleteness_Subclade_wraf124.pdf]

A

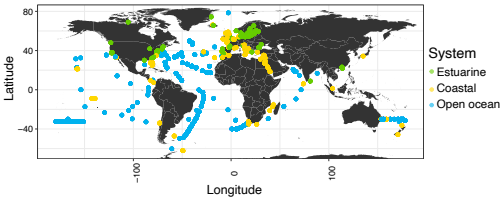

B

Open ocean  
Welch Anova,  $p = <0.0001$

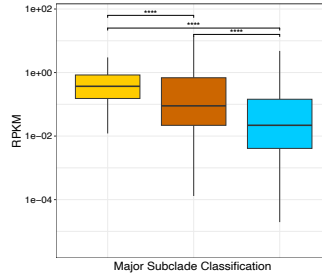

C

Coastal  
Welch Anova,  $p = <0.0001$

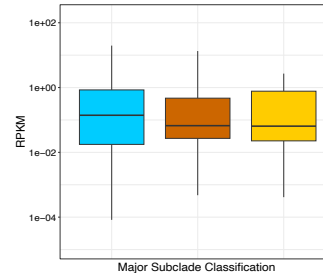

D)

Estuarine  
Welch Anova,  $p = 0.25$

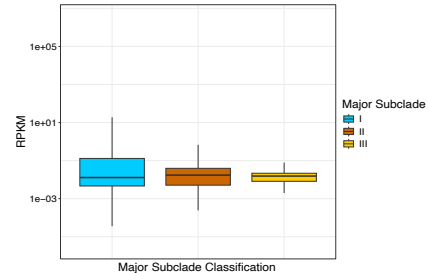

Supplement: supplementary-material_wraf124 [file supplementary-material_wraf124.zip › FigureS5_System_Types_MajorSubclade_wraf124.pdf]

# Linear regression of salinity vs Subclade RPKM

Open ocean, coastal, and estuarine systems

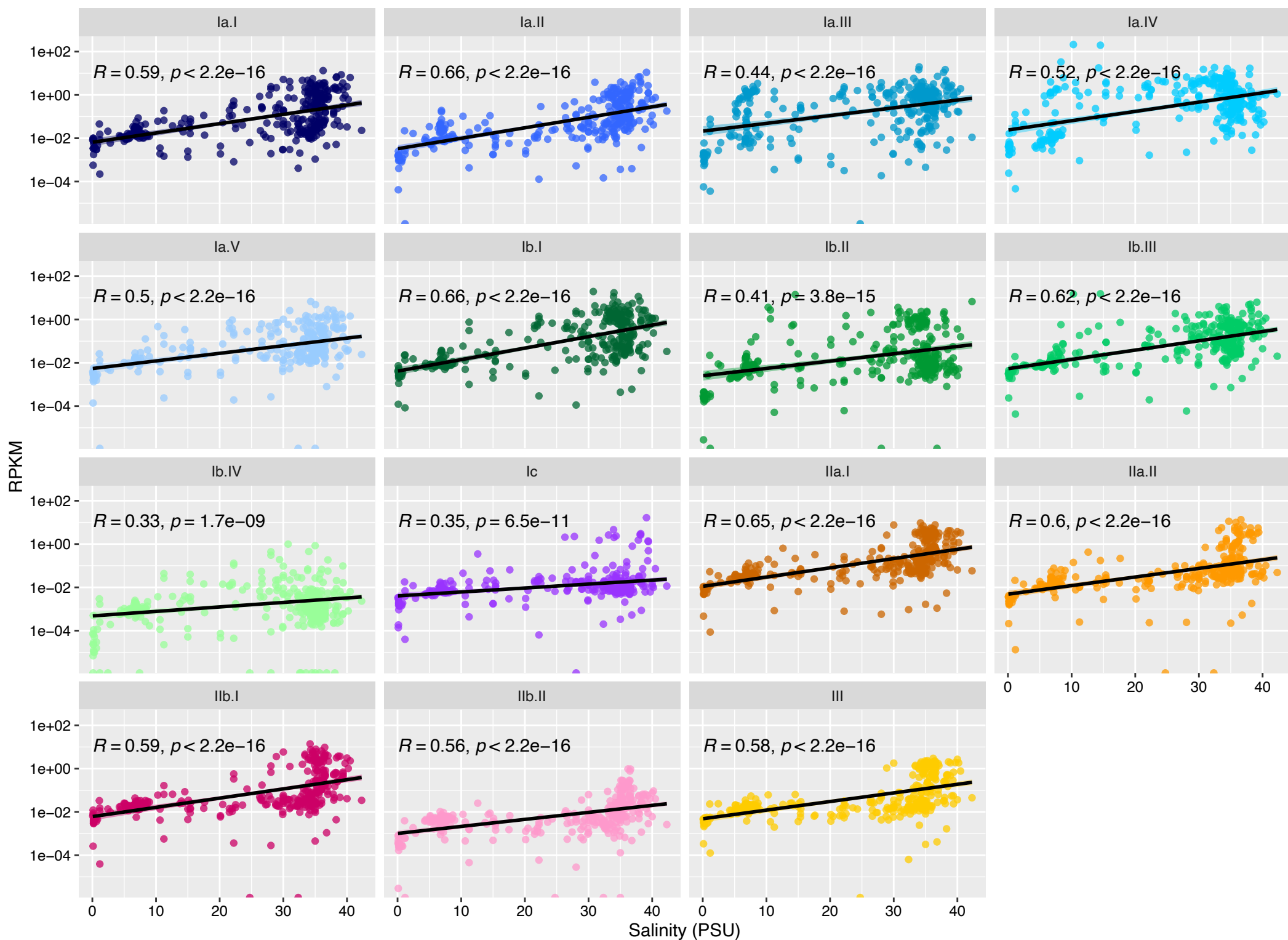

Supplement: supplementary-material_wraf124 [file supplementary-material_wraf124.zip › FigureS6_LinearRegressionALL_Salinity_wraf124.pdf]

# Linear regression of salinity values vs Subclade RPKM

Estuarine systems

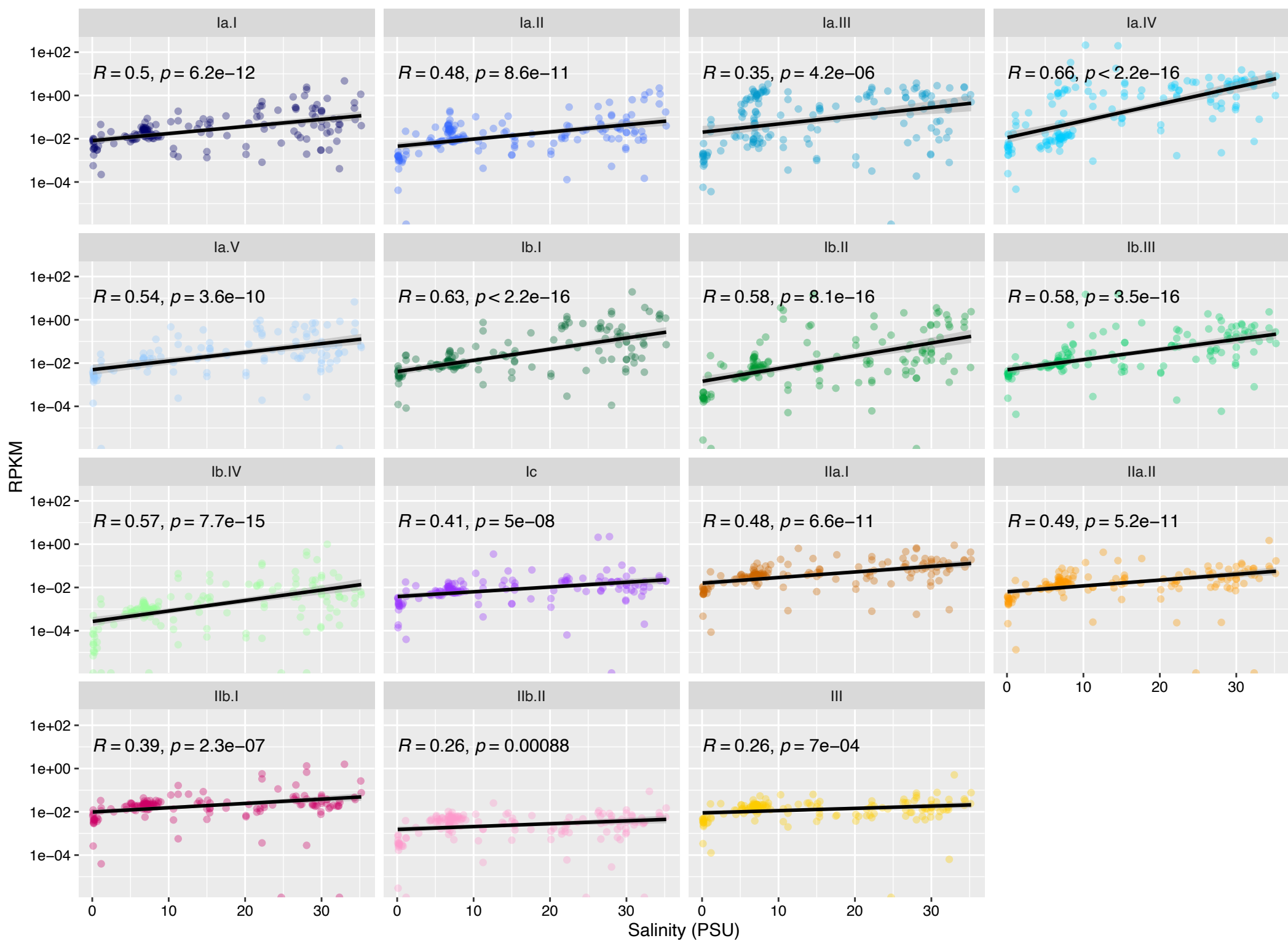

Supplement: supplementary-material_wraf124 [file supplementary-material_wraf124.zip › FigureS7_LinearRegression_Sal_Estuarine_wraf124.pdf]

# Linear regression of salinity values vs Subclade RPKM

Coastal systems

RPKM

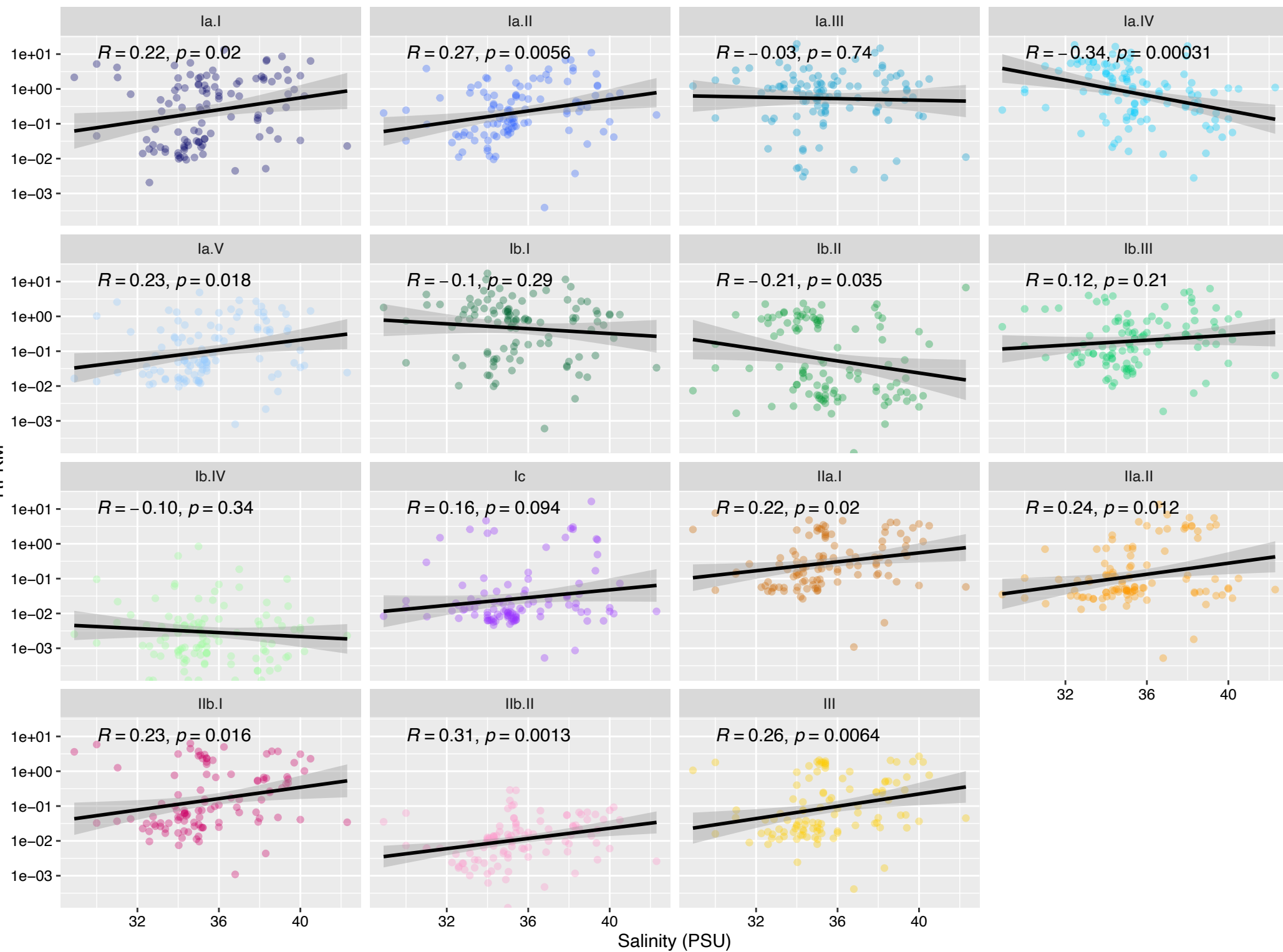

Supplement: supplementary-material_wraf124 [file supplementary-material_wraf124.zip › FigureS8_LinearRegression_Sal_Coastal_wraf124.pdf]

# Linear regression of salinity values vs Subclade RPKM

Open ocean systems

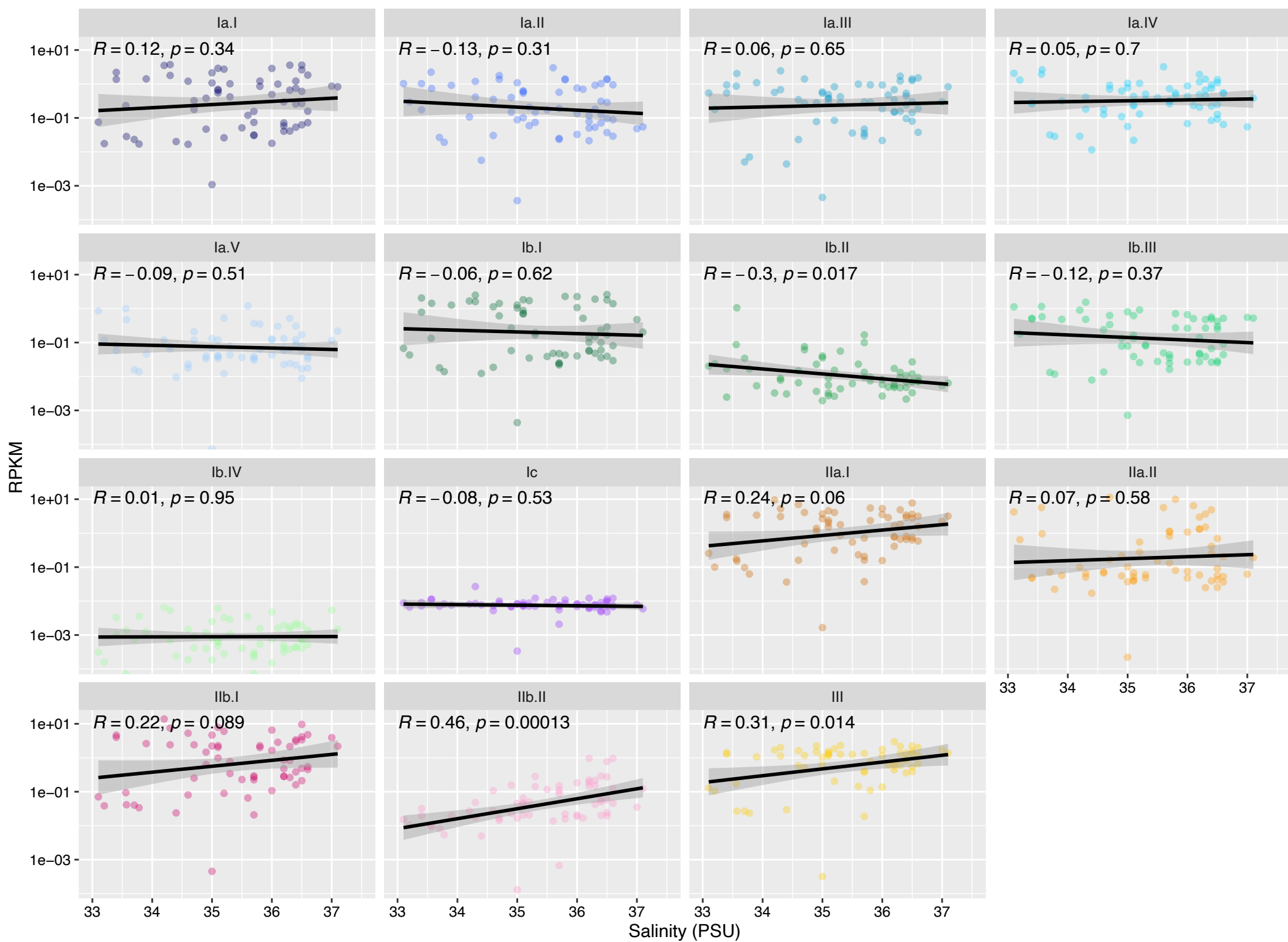

Supplement: supplementary-material_wraf124 [file supplementary-material_wraf124.zip › FigureS9_LinearRegression_Sal_OpenOcean_wraf124.pdf]
