# Supplementary material for: Culture-supported ecophysiology of the SAR116 clade demonstrates metabolic and spatial niche partitioning
Source: ISME J. 2025 Jun 13;19(1):wraf124. doi: 10.1093/ismejo/wraf124 (PMC12262181; doi:10.1093/ismejo/wraf124)
Supplement: supplementary-material_wraf124 [file FigureS17_LSUCC0744_size_panel_wraf124.pdf › FigureS17_LSUCC0744_size_panel_wraf124.pdf]

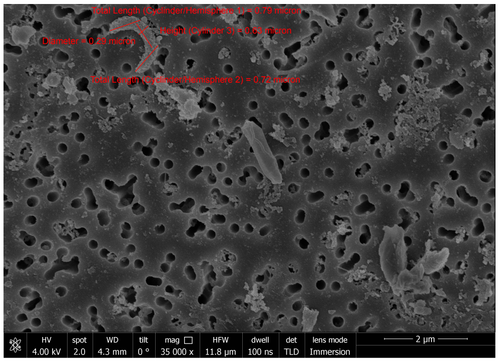
Total Length (Cyclinder/Hemisphere 1) = 0.79 micron
Height (Cylinder 3) = 0.63 micron
Diameter = 0.29 micron
Total Length (Cyclinder/Hemisphere 2) = 0.72 micron
HV 4.00 kV
spot 2.0
WD 4.3 mm
tilt 0 °
mag 35 000 x
HFW 11.8 µm
dwell 100 ns
det TLD
lens mode Immersion
2 µm

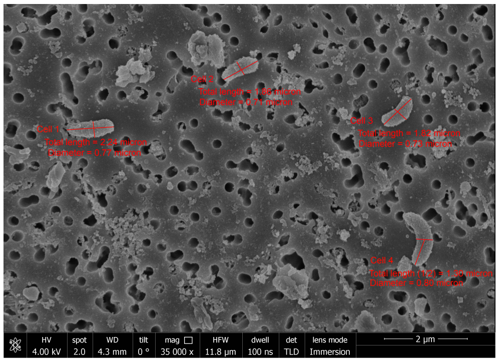
Cell 2
Total length = 1.86 micron
Diameter = 0.71 micron
Cell 1
Total length = 2.24 micron
Diameter = 0.77 micron
Cell 3
Total length = 1.82 micron
Diameter = 0.73 micron
Cell 4
Total length (1/2) = 1.30 micron
Diameter = 0.80 micron
HV 4.00 kV
spot 2.0
WD 4.3 mm
tilt 0 °
mag 35 000 x
HFW 11.8 µm
dwell 100 ns
det TLD
lens mode Immersion
2 µm

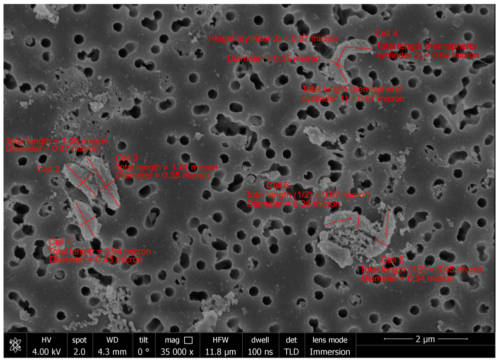
Height (cylinder 3) = 0.37 micron
Cell 4
Total length (hemisphere/ cyclinder 2) = 0.64 micron
Diameter = 0.35 micron
Total length (hemisphere/ cyclinder 1) = 0.57 micron
Total length = 1.05 micron
Diameter = 0.47 micron
Cell 3
Total length = 1.41 micron
Diameter = 0.35 micron
Cell 2
Cell 6
Total length (1/2) = 0.67 micron
Diameter = 0.38 micron
Cell 1
Total length = 0.94 micron
Diameter = 0.43 micron
Cell 5
Total length (1/2) = 0.65 micron
Diameter = 0.34 micron
HV 4.00 kV
spot 2.0
WD 4.3 mm
tilt 0 °
mag 35 000 x
HFW 11.8 µm
dwell 100 ns
det TLD
lens mode Immersion
2 µm

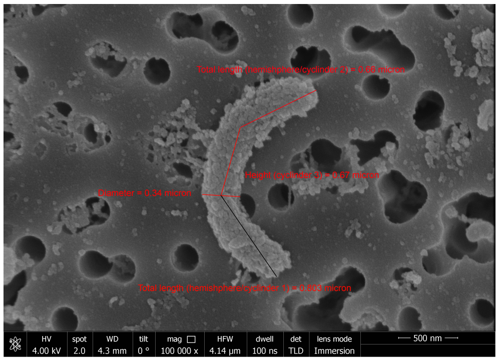
Total length (hemishphere/cyclinder 2) = 0.66 micron
Height (cyclinder 3) = 0.67 micron
Diameter = 0.34 micron
Total length (hemishphere/cyclinder 1) = 0.803 micron
HV 4.00 kV
spot 2.0
WD 4.3 mm
tilt 0 °
mag 100 000 x
HFW 4.14 µm
dwell 100 ns
det TLD
lens mode Immersion
500 nm

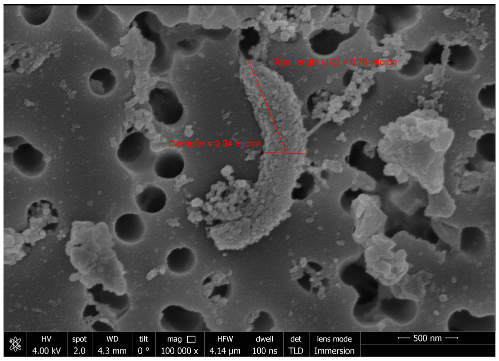
Total length (1/2) = 0.76 micron
Diameter = 0.34 micron
HV 4.00 kV
spot 2.0
WD 4.3 mm
tilt 0 °
mag 100 000 x
HFW 4.14 µm
dwell 100 ns
det TLD
lens mode Immersion
500 nm

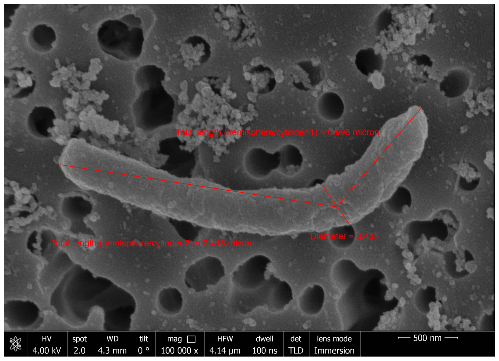
Total length (hemisphere/cylinder 1) = 0.996 micron
Diameter = 0.435
Total length (hemisphere/cylinder 2) = 2.418 micron
HV 4.00 kV
spot 2.0
WD 4.3 mm
tilt 0 °
mag 100 000 x
HFW 4.14 µm
dwell 100 ns
det TLD
lens mode Immersion
500 nm

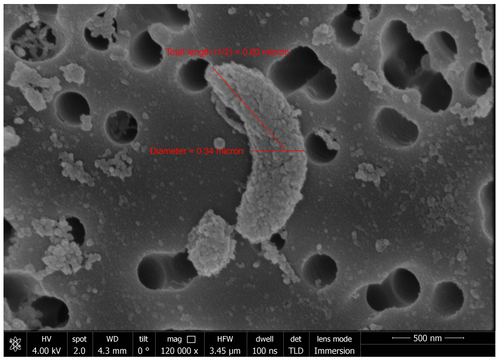
Total length (1/2) = 0.60 micron
Diameter = 0.34 micron
HV 4.00 kV
spot 2.0
WD 4.3 mm
tilt 0 °
mag 120 000 x
HFW 3.45 µm
dwell 100 ns
det TLD
lens mode Immersion
500 nm

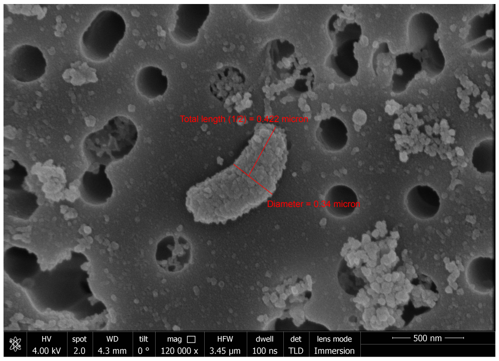
Total length (1/2) = 0.422 micron
Diameter = 0.34 micron
HV 4.00 kV
spot 2.0
WD 4.3 mm
tilt 0 °
mag 120 000 x
HFW 3.45 µm
dwell 100 ns
det TLD
lens mode Immersion
500 nm

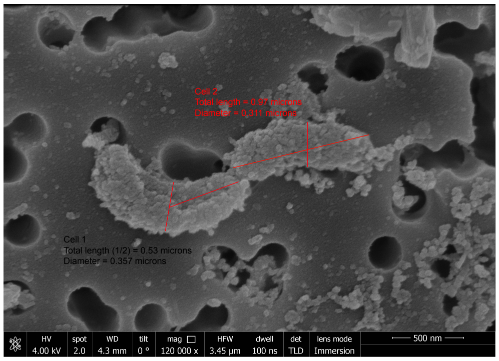
Cell 2
Total length = 0.97 microns
Diameter = 0.311 microns
Cell 1
Total length (1/2) = 0.53 microns
Diameter = 0.357 microns
HV 4.00 kV
spot 2.0
WD 4.3 mm
tilt 0 °
mag 120 000 x
HFW 3.45 µm
dwell 100 ns
det TLD
lens mode Immersion
500 nm
