# Supplementary material for: Culture-supported ecophysiology of the SAR116 clade demonstrates metabolic and spatial niche partitioning
Source: ISME J. 2025 Jun 13;19(1):wraf124. doi: 10.1093/ismejo/wraf124 (PMC12262181; doi:10.1093/ismejo/wraf124)
Supplement: supplementary-material_wraf124 [file FigureS1_ANI_AAI_Phyotree_wraf124.pdf › FigureS1_ANI_AAI_Phyotree_wraf124.pdf]

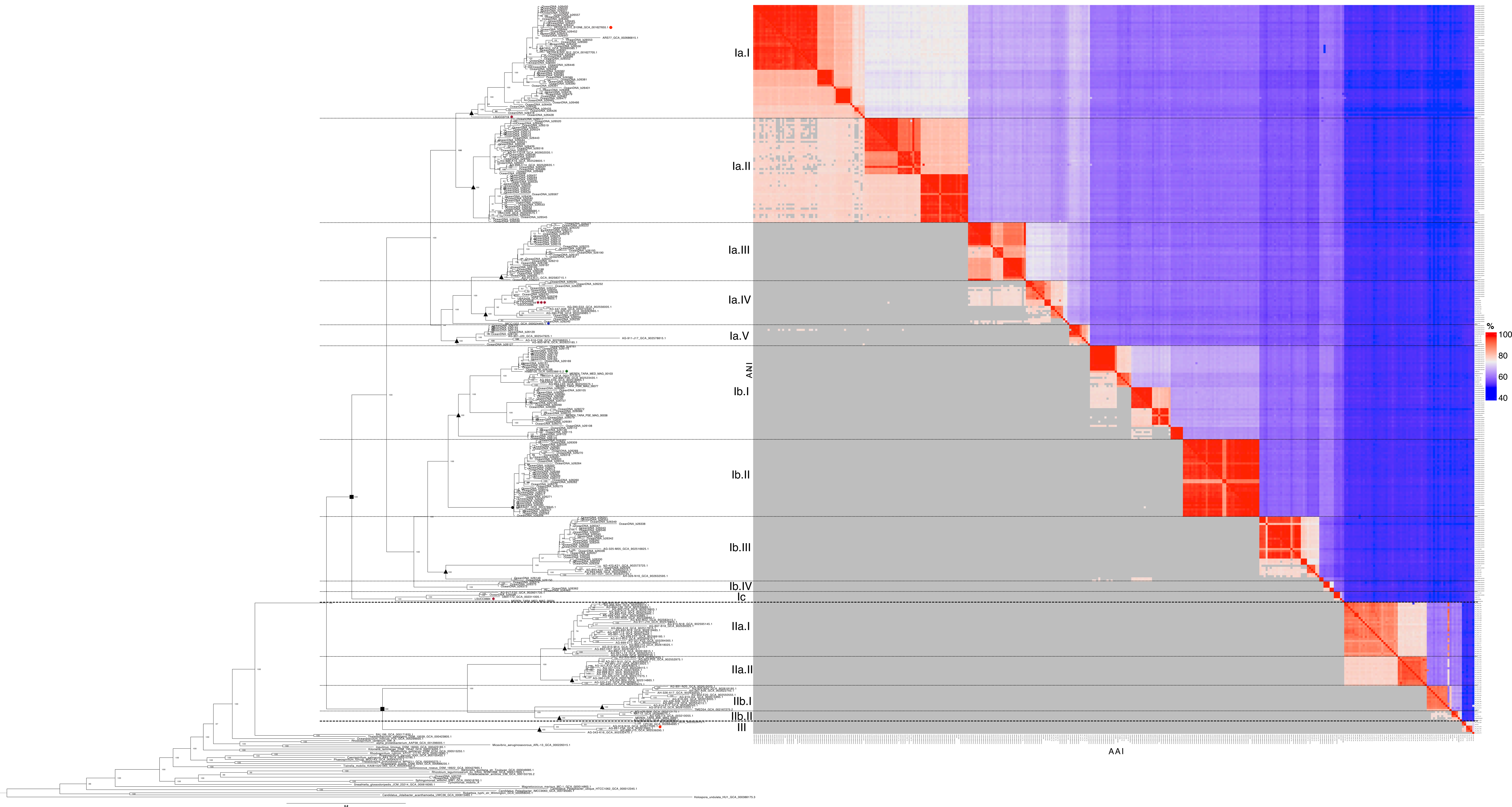

Ia.I
Ia.II
Ia.III
Ia.IV
Ia.V
Ib.I
Ib.II
Ib.III
Ib.IV
Ic
IIa.I
IIa.II
IIb.I
IIb.II
III
ANI
AAI
%
100
80
60
40
