# Supplementary material for: Culture-supported ecophysiology of the SAR116 clade demonstrates metabolic and spatial niche partitioning
Source: ISME J. 2025 Jun 13;19(1):wraf124. doi: 10.1093/ismejo/wraf124 (PMC12262181; doi:10.1093/ismejo/wraf124)
Supplement: supplementary-material_wraf124 [file FigureS3_KEGG-Decoder_raw_output_wraf124.pdf › FigureS3_KEGG-Decoder_raw_output_wraf124.pdf]

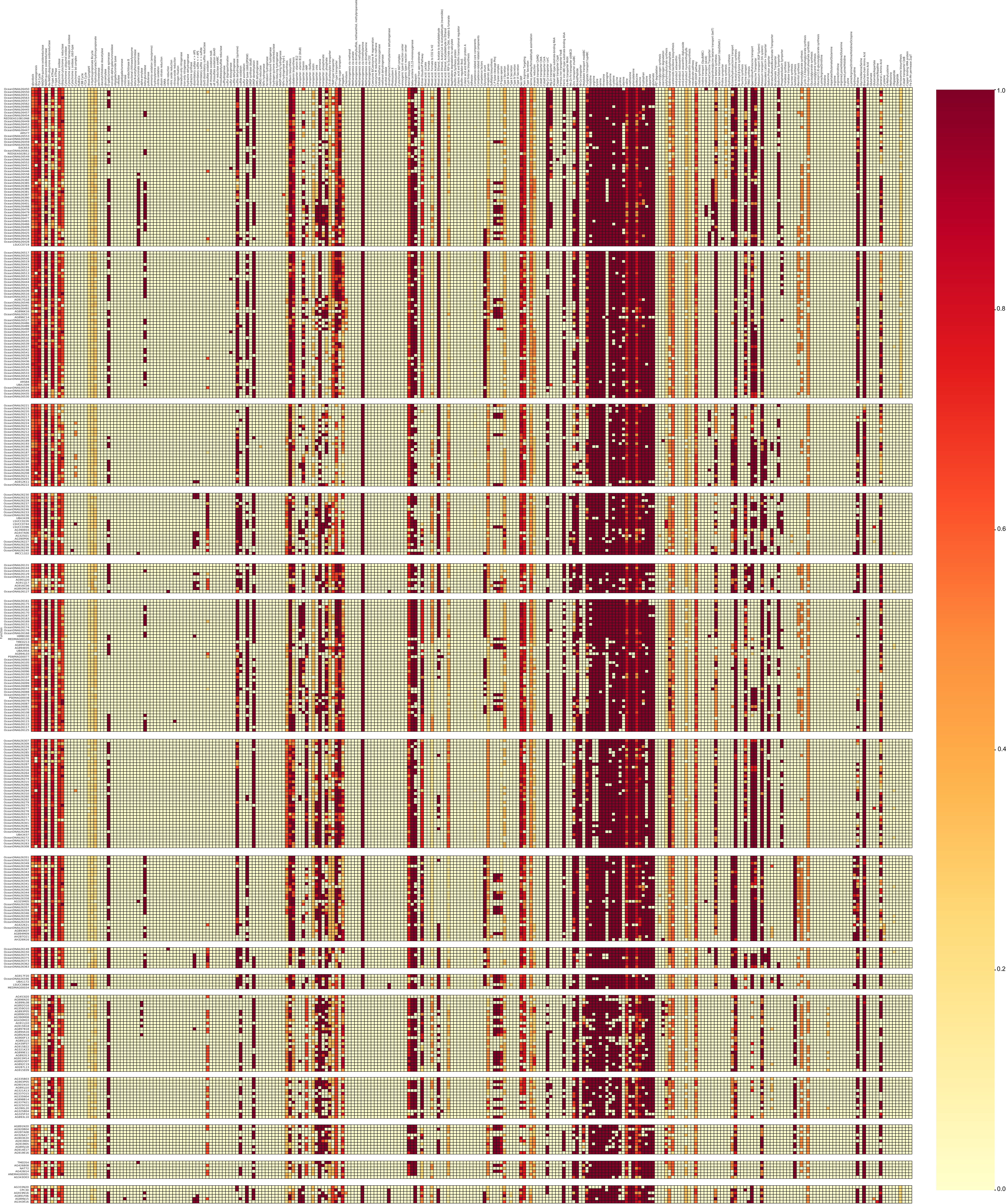
1.0
0.8
0.6
0.4
0.2
0.0
